# A Mouse Model of PPRV Infection for Elucidating Protective and Pathological Roles of Immune Cells

**DOI:** 10.3389/fimmu.2021.630307

**Published:** 2021-04-12

**Authors:** Yashu Sharma, Roman Sarkar, Ayush Jain, Sudhakar Singh, Chander Shekhar, Chandrasekar Shanmugam, Muthuchelvan Dhanavelu, Prabhakar Tembhurne, Rajeev Kaul, Sharvan Sehrawat

**Affiliations:** ^1^ Department of Biological Sciences, Indian Institute of Science Education and Research Mohali, Mohali, India; ^2^ Division of Virology, Indian Veterinary Research Institute, Mukteshwar, India; ^3^ Department of Veterinary Microbiology, Nagpur Veterinary College, Nagpur, India; ^4^ Department of Microbiology, University of Delhi, New Delhi, India

**Keywords:** peste des petits ruminants virus, CD8 T cells, mouse model, class I epitopes, pathogenicity, IFNR KO

## Abstract

The study was aimed at developing an accessible laboratory animal model to elucidate protective and pathological roles of immune mediators during Peste des petits ruminants virus (PPRV) infection. It is because of the critical roles of type I IFNs in anti-viral defense, we assessed the susceptibility of IFN receptor knock out (IFNR KO) mice to PPRV infection. IFNR KO mice were exceedingly susceptible to the infection but WT animals efficiently controlled PPRV. Accordingly, the PPRV infected IFNR KO mice gradually reduced their body weights and succumbed to the infection within 10 days irrespective of the dose and route of infection. The lower infecting doses predominantly induced immunopathological lesions. The viral antigens as well as the replicating PPRV were abundantly present in most of the critical organs such as brain, lungs, heart and kidneys of IFNR KO mice infected with high dose of the virus. Neutrophils and macrophages transported the replicating virus to central nervous system (CNS) and contributed to pathology while the elevated NK and T cell responses directly correlated with the resolution of PPRV infection in WT animals. Using an array of fluorescently labeled H-2K^b^ tetramers, we discovered four immunogenic epitopes of PPRV. The PPRV-peptides interacted well with H-2K^b^ in acellular and cellular assay as well as expanded the virus-specific CD8^+^ T cells in immunized or infected mice. Adoptively transferred CD8^+^ T cells helped control PPRV in infected mice. Our study therefore established and employed a mouse model for investigating the pathogenesis of PPRV. The model could be useful for elucidating the contribution of immune cells in disease progression as well as to test anti-viral agents.

## Introduction

Peste des petits ruminants virus (PPRV) causes high mortality in herds of small ruminants and is responsible for major economic losses to livestock sector in developing countries (1–4). PPRV is a negative sense, single stranded enveloped virus of paramyxoviridae family that also harbors several other members able to cause debilitating disease in animals and humans. Some of the examples are Rinderpest virus (RPV) and Canine distemper virus (CDV) of animals and measles virus (MeV) and mumps virus (MuV) of humans (5, 6). PPRV genome encodes for six structural proteins i.e., nucleocapsid (N), phosphoprotein (P), matrix (M), fusion (F), hemagglutinin (H) and polymerase (L) and two nonstructural proteins, C and V (5). The protective and pathological mechanisms activated by PPRV as well as the contribution of immune cells in the viral pathogenesis have not been adequately investigated. This could primarily be attributed to the unavailability of an accessible laboratory animal model. We, therefore, addressed this issue in the current investigation to develop a more accessible laboratory animal model to better understand immunity and immunopathology following PPRV infection.

Currently, a live attenuated prophylactic vaccine against PPRV is used in small ruminants. While the vaccine induces a lasting immunity, a transient immunosuppression is usually evident in vaccinated animals and this could enhance their susceptibility to heterologous infections (3, 5). Therefore, it is imperative to assess cellular and molecular mediators induced in animals following PPRV infection or vaccination. Such investigations could shed light on immune correlates of host protection and help devise improved vaccination strategies. This endeavor is even more relevant for the contemporary animal health care system as elaborate efforts are made to eradicate PPRV globally. That an intensive vaccination program could help eradicate PPRV is bolstered by the achieved success in eradicating a related virus, RPV. Therefore, an accessible laboratory animal model would likely improve understanding of PPRV pathogenesis.

We studied viral pathogenesis in mice ablated of IFN response and observed enhanced susceptibility of IFN receptor knockout (IFNR KO) mice to PPRV infection. The infected animals succumbed to it within 10 days. The inoculum size however altered the nature of resulting pathology. While higher doses of PPRV predominantly induced a virus-mediated disease, an immunopathological manifestation was evident at lower doses. The replicating PPRV as well as the viral antigens were abundant in most of the analyzed organs. Innate immune cells such as neutrophils and macrophages likely transported the replicating PPRV to brain tissues. We then identified immunogenic class I (H-2K^b^) restricted epitopes of PPRV in mice using a combination of *in silico*, *ex vivo* and *in vivo* approaches. Adoptively transferred WT CD8^+^ T cells isolated from PPRV infected or the peptide immunized mice conferred survival advantage to the infected IFNR KO mice. Therefore, our study established a laboratory animal model that could be valuable for understanding the immunological and virological parameters in morbillivirus pathogenesis.

## Materials and Methods

### Mice

C57BL/6J (Stock no- 000664), IFNR KO (B6.Cg-*Ifngr1^tm1Agt^ Ifnar^1tm1.2Ees^*/J; Stock no- 029098) and B6 CD45.1 (B6.SJL-*Ptprc^a^ Pepc^b^*/BoyJ; Stock no- 002014) mice were procured from Jackson Laboratory USA. All the animals were housed and bred in the individual ventilated cages in the small animal facility for experimentation of Indian Institute of Science Education and Research (IISER), Mohali. Six to eight weeks old mice were used for performing all the experiments. The animal experiments were performed strictly in accordance with the protocol approved by the Institutional Animal Ethics Committee (IAEC), IISER Mohali, constituted under the aegis of committee for the purpose of control and supervision of experiments on animals (CPCSEA).

### Virus and Cell Lines

PPRV vaccine strain Sungri/96 was used for all the *ex vivo* and *in vivo* experiments. The virus was cultured, harvested and titrated using Vero cells and stored at -80˚C till further use as described earlier (4, 7). The infecting dose of the virus was calculated as TCID_50_ values as well as plaque forming units (PFU) by a previously described method (8). RAW264.7 cells and RMA/S cells were purchased from National Centre for Cell Science, Pune, India. The cells were cultured in RPMI (Gibco‐BRL, Rockville, MD, USA) supplemented with 10% Fetal bovine Serum (FBS), penicillin (100U/mL) and streptomycin (100μg/mL) in a humidified CO_2_ incubator.

### Antibodies and Other Biological Reagents

Antibodies used in this study were purchased from BD Biosciences, Tonbo biosciences, BioLegend, and eBiosciences. The antibodies used were against CD4-PE and FITC (clone GK1.5), purified CD16/32 (Clone 2.4G2), CD11b-FITC (clone M1/70), Gr1-APC (clone RB6-8C5), F4/80-PE (clone T45-2342), CD8-PerCP Cy5.5 and PE (clone 53-6.7), H2K^b^-FITC (clone AF6 88.5), mouse IgG-FITC, CD45.1-APC (clone A20), CD45.2-FITC (clone 1O4), CXCR3-FITC (clone 173), CD44-APC (clone IM7), CD62L-APC (clone MEL 14), NK1.1-PE-Cy7 (clone PK136) and CD45-PerCP Cy5.5 (Clone 30-F11). All the antibodies were diluted in FACS buffer (Phosphate buffered saline (PBS) with 2% FBS) in 1:100 ratios. For staining of innate immune cells upon PPRV infection in mice, antibody mix was made with anti-CD45-PerCP Cy5.5, anti-CD11b-FITC, anti-F4/80-PE, anti-NK1.1-PE Cy7 and anti-Gr1-APC. For analyzing T cells response, anti-CXCR3-FITC, anti-CD4-PE, anti-CD8-PerCP Cy5.5 and anti-CD62L-APC were combined. To measure T cells in non-lymphoid organs, antibody combinations included anti-CD45-PerCP Cy5.5, anti-CD4-FITC and anti-CD8-PE.

Other reagents such as Dulbecco’s modified essential medium (DMEM), RPMI 1640, FBS and penicillin-streptomycin antibiotics were purchased from Gibco, BRL, Rockville, MD, USA. Trypsin, SYBR Green and propidium iodide were obtained from Life Technologies. Hematoxylin Eosin Y (H&E) was purchased from HiPrep, M-CSF was purchased from Peprotech and optimal cutting temperature (OCT) compound was obtained from Fisher Health Care. *p*-nitrophenol phosphate (*p*-NPP) and Freund’s complete and incomplete adjuvants were procured from Sigma-Aldrich.

### Generation of Bone Marrow Derived Macrophages (BMDMs)

C57BL/6 mice were sacrificed by terminally anesthetizing with CO_2_ and sprayed with 70% alcohol. Long bones were dislocated and skin as well as muscles were gently removed. Thereafter, the bones were sterilized by dipping in 70% alcohol. To collect bone marrow cells, the bones were flushed by using cold RPMI medium and single cell suspensions were prepared as described earlier (9). RBCs were lysed using RBC lysis buffer (155mM NH_4_Cl, 12mM NaHCO_3_ and 0.1mM EDTA, pH 8.0). The bone marrow cells were analyzed for live dead counts and were then cultured in 24 well plates (1x10^6^ cells/well) in the presence of 10ng/ml M-CSF for 7 days in a humidified 5%CO_2_ incubator. Medium was replaced after every two days. After 7 days, cells were collected, washed and used for performing further experiments.

### Measuring Type I and Type II IFN Response in PPRV Pulsed RAW264.7 Macrophages Cell Line and BMDMs by qRT-PCR

To measure type I and II interferon response in RAW macrophages and BMDMs, 2x10^5^ cells were incubated in complete RPMI in a 24-well plate with live or heat inactivated PPRV (*i*-PPRV) at 1 and 10 MOI for 15 min, 30 min, 1hr and 6hr. The control cells were either added with the lysates from Vero cells or were left untreated. The cells were harvested and washed with PBS at the indicated time points. Total RNA was isolated from PPRV pulsed and control cells as described earlier (10). The isolated total RNA was then converted into cDNA using a first strand cDNA synthesis kit (Verso cDNA synthesis kit, Thermo Fisher Scientific) according to the manufacturer’s protocol. Qualitative real time polymerase chain reaction (qRT-PCR) was carried out using 2x-DyNamo ColorFlash SYBR Green qRT-PCR kit (ThermoFisher) as per the manufacturer’s protocol in a QuantStudio Real Time PCR system (ThermoFisher). The expression of hypoxanthine phosphoribosyltransferase 1 (HPRT 1) gene served as an internal control. To calculate fold change in gene expression, we first normalized the values with the internal control (HPRT1) to obtain ΔCt values. This ΔCt values were then normalized with the experimental control to obtain Δ(Δ(tain zeds. The relative expression for each gene was then calculated by using 2^-Δ(ΔCt)^ values as described elsewhere (11). The sequence of primers and the amplicon size are described in **Table S1**.

### Infection of Mice With PPRV and Collection of Organs

Animals were infected with the indicated doses of PPRV using intraperitoneal (*i.p.*) or intranasal (*i.n.*) routes. Different physiological parameters such as body weight, behavior and the survival rates were measured in different groups of animals. For most of the experiments, the animals were sacrificed at the termination of experiments when body weights dropped by more than 20% in any of the experimental groups. Different lymphoid and non-lymphoid organs were collected to detect the presence of replicating virus, the viral antigens, and for cellular analysis in lymphoid organs such as spleen, mediastinal LNs as well as non-lymphoid organs such as lungs, brain tissues and in the bronchoalveolar lavage (BAL) fluids. Before collecting organs from different groups of mice, animals were perfused *via* intracardiac injection with 20ml of PBS in left ventricle after making an incision in the draining blood vessels posterior to the diaphragm to remove any contaminating blood cells from the collected organs (12, 13).

### Flow Cytometry for Cellular Analysis

Different lymphoid and non-lymphoid organs were collected from infected or immunized mice. To prepare single cell suspension from lymphoid organs, the organs were placed in 70μm cell strainer with 5ml of cold complete RPMI and gently crushed using the soft end of a 2.5ml syringe plunger. All the suspended cells were passed through cell strainer and collected in a 15ml tube. These cells were then washed with PBS by centrifugation at 1200rpm for 5 min at 4°C. After three washings, the cells were finally resuspended in cold complete RPMI and counted using a hemocytometer for further cellular analysis. The single cell suspensions were prepared from the collected non-lymphoid organs by digesting with type IV collagenase as described earlier (14). 1x10^6^ cells were stained using indicated fluorescent labeled antibodies at 4**°**C for 30 minutes. Fc block (anti-CD16/32 antibody) was performed before surface staining. For intracellular staining, first cells were surface stained and then fixed with 4% paraformaldehyde for 20 min at 4**°**C. Thereafter the cells were permeabilized using permeabilization buffer from eBioscience. The cells were then incubated with Fc block and antibodies against PPRV viral antigens (H or N) for 30 min. After washing three times with PBS, cells were stained with fluorescently labeled anti-mouse IgG antibody for 30 min at 4**°**C. The stained cells were washed three times and acquired using a BD Accuri C6 or BD FACS Aria fusion. The analysis of the data was performed by Flow Jo software v10.

### Cell Purification and Adoptive Transfer

Different subsets of T cells either from naïve or previously PPRV infected C57BL/6 mice were purified either by magnetic cell sorting or by FACS. Similarly, innate immune cells such as macrophages (CD11b^+^F4/80^+^), neutrophils (CD11b^+^Gr1^hi^) and dendritic cells (CD11b^+^CD11c^+^) from CD45.1^+^ mice were purified. The sorted cells were collected at low temperature in complete RPMI. The cells were then pulsed with 1MOI of PPRV for one hour at 37**°**C. After extensive washings, the innate immune cells were counted and analyzed for the presence of PPRV antigens. 1x10^6^ of PPRV pulsed innate immune cells were adoptively transferred in IFNR KO mice (CD45.2^+^) intravenously (n=3 mice per group). One group of animals that did not receive any cells was *i.p* infected by PPRV with 10^5^ PFU/mouse. The disease progression and body weight change were monitored subsequently.

### Reconstitution of IFNR KO Mice With T Cells to Measure Their anti-PPRV Functions

In order to measure the protective ability of immune cells against PPRV, CD8^+^ T cells were MACS purified from naïve C57BL/6 WT mice or from WT mice previously infected with PPRV (5x10^6^ PFU) for 5 days. The purified cells were adoptively transferred in sex matched IFNR KO animals. The recipient animals were subsequently infected with PPRV. Mice that did not receive any cells served as the controls. All animals were observed for change in body weights and survival. At the termination of experiments, lymphoid and non-lymphoid organs of animals were collected for cellular analysis. In another experiment, 1x10^6^ MACS purified CD4^+^ and CD8^+^ T cells from naïve mice were adoptively transferred in sex matched naïve IFNR KO mice and the cells were allowed to proliferate under lymphopenic environment to reconstitute a pool of WT cells. After 40 days, the IFNR KO mice were infected with 1 PFU of PPRV. The disease progression and change in body weight were monitored in these animals before termination of the experiment at 6dpi.

### Isolation of Inflammatory Cells From Brain Tissues, Lungs and Bronchoalveolar Lavage Fluid

Inflammatory cells were isolated from the brain and lung tissues of IFNR KO mice using protocol as described earlier (15). Briefly, brain tissues were minced into 3-4mm pieces with a sterile scalpel or scissors under complete aseptic conditions. Washing was done 4-5 times with 10mM PBS. Part of tissue pieces were stored for sectioning and to the remainder of tissues a 0.25% trypsin solution prepared in RMPI was added. The samples were then incubated at 4**°**C for 16 hrs. Thereafter, trypsin solution was removed and the samples were incubated at 37**°**C for 30 min. Complete RPMI was added to prepare single cell suspension. After washing with PBS, cell suspensions were used for further analysis. BAL fluids from control and *i.n* infected mice with PPRV were collected as described elsewhere (16).

### Fluorescent Microscopy and Histological Analysis

The organs collected from control and PPRV infected animals were first fixed in 4% paraformaldehyde overnight followed by their dehydration with a gradient of 5%-20% sucrose solution at room temperature. The dehydrated organs were embedded in OCT compound to form blocks and stored at -80**°**C for sectioning. 6μm tissue sections were cut by a cryotome and the sections were dried and used for immunostaining. For performing immunostaining, tissue sections were first blocked using anti-CD16/CD32 antibody. The sections were then incubated with anti-PPRV (H) and (N) monoclonal antibodies. After washing, these sections were stained with anti-mouse IgG-FITC antibody and visualized using a fluorescent microscope. The images were analyzed by using Image J software. For hematoxylin and eosin Y (H and E) staining, the tissue section from brain tissues were air dried and stained. The sections were visualized and images were recorded using Leica DMi8 microscope as described earlier (14).

### Bioinformatic Analysis to Predict Immunogenic Peptides of PPRV

Amino acid sequences of PPRV structural proteins were retrieved from National Centre for Biotechnology Information (NCBI) database in FASTA Format. The immunogenic peptides for one of the class I MHC molecules of C57BL/6 mice (H-2K^b^), were predicted using immune epitope database (IEDB) and analysis resource. The software uses artificial network (ANN) and stabilized matrix method (SMM). The percentile rank of <2 and IC_50_ values were used for predicting peptides. A low percentile rank and the IC_50_ values lower than 50nM indicated high affinity binders. The peptides with IC_50_ values between 50 and <500nM were considered as intermediate affinity binders while those with >500nM were considered as the low affinity binders (17, 18). Additional parameter for selecting the peptides were their immunogenicity scores (17, 18). A percentile rank for the predicted peptides was generated by comparing IC_50_ values of predicted epitopes against a set of random peptides using SWISSPROT database. The selected peptides were commercially synthesized and obtained from GL Biochem. The purity of the synthetic peptides was greater than 90%.

### Molecular Docking Analysis

For predicting the binding affinities of different peptides with class I MHC molecules (H–2K^b^ and caprine leukocyte antigen, CLA1), molecular docking analyses were performed using HPEPDOCK–web server. Default parameters were used for all the docking analysis as described earlier (17). HPEPDOCK server uses a hierarchical algorithm, MODPEP program generates protein–peptide docking in a blind fashion to yield an ensemble of peptide conformation for each peptide. The PDB File 1S7Q was used for homology modeling of peptides with H–2K^b^ protein. To test the efficiency of docking algorithm, a known immunogenic 9–mer peptide, FAPGNYPAL (derived from Sendai E virus (SEV) nucleoprotein, was used in context with H–2K^b^. We then compared the energy parameters of the best–selected structures among different PPRV peptides docked with H–2K^b^. To further refine and define the interacting residues both quantitatively and qualitatively, we used molecular modeling program UCSF Chimera for binding analysis (19). As the goal of such experiments was to explore the translational value of such peptides in small ruminants, we superimposed H–2K^b^ with goat class I MHC (CLA–1) molecule at 1.5A RMSD (root mean square distance) using the tool, Matchmaker, available in the UCSF Chimera. Similarly, docking studies were performed for CLA–I with different PPRV–peptides and representative docked structures were generated using UCSF Chimera. A comparative analysis between docking scores of H–2K^b^ and CLA–I for the similar peptides was also performed.

### Class I MHC Stabilization Assays

The stabilization of class I MHC by each peptide was measured using both cellular and acellular assays. TAP deficient murine T cell lymphoma cells (RMA/S cells) were used for determining the peptide induced surface stabilization of class I MHC molecule by flow cytometric analysis as described earlier (20). RMA/S cells were maintained in complete RPMI medium. 2x10^5^ cells were serum starved for 3 hrs at 37**°**C and subsequently pulsed with the respective peptides to induce their surface class I MHC (H–2K^b^) stabilization. Graded doses of peptides were added to the cells in serum free RPMI and the cells were incubated at 37**°**C for 7 hrs. The cells were then washed with PBS and stained with an anti–H–2K^b^ antibody. Live and dead cells were differentiated using propidium iodide (PI) staining and gated live cells were measured for the expression of H–2K^b^. The cells were analyzed by FACS Accuri. The obtained data were represented as percent positive cells for the expression of H–2K^b^. EC_50_ value for high affinity peptides were then calculated.

For determining the class I MHC stabilization, an acellular assay described elsewhere was also used (21). Briefly, ELISA plates were coated with streptavidin overnight at 4**°**C. Subsequently, H–2K^b^ monomers were added to the plates. The monomers were generated by refolding a UV photocleavable ligand, (FAPG(Anp)YPAL), β2 microglobulin and H–2K^b^ heavy chain followed by their biotinylation as described earlier (20, 22). The unbound H–2K^b^ monomers were washed off. Control and PPRV–peptides were added to the identified wells in the plates. The plates were then exposed to UV radiations at 365nM for 30 min to achieve the displacement of UV ligand with respective peptides. The efficiency of exchange was measured by probing the washed plates by adding anti–β2 microglobulin antibody. Subsequently, a mouse anti–IgG antibody enzyme conjugate (1:10, 000) was added after washing the plates. Thereafter, substrate, (p–nitrophenol phosphate (1mg/ml) was added for its conversion into a chromogenic product. The stop solution was used to block the reaction and the plates were measured for absorbance at 405nm. Positive and negative controls were also included in the study as described earlier (23).

### PPRV Infection and PPRV–Peptide Immunization of Mice for Measuring Virus–Specific CD8^+^ T Cells

In order to determine the immunogenicity of predicted peptides *in vivo*, we performed two types of experiments. Throughout the manuscript, the terms plaque forming units (PFU) and focal forming units (FFU) are used interchangeably as clear plaques were not observed when PPRV was grown in Vero cells. In first set of experiments, WT C57BL/6 mice were *i.p.* infected with a high dose of PPRV (5x10^6^ PFU). After seven days, a second dose of PPRV was given to animals to boost responses. The analysis of the expanded cells was performed three days later by measuring the frequencies PPRV–peptide specific CD8^+^ T cells by tetramer staining. In second set of experiments, C57BL/6 mice were immunized with the cocktail of PPRV–peptides (AILTFLFLL, FMYLFLLGV, FSAGAYPLL and IGLVRDFGL) prepared in complete Freund’s adjuvant. Mice were immunized subcutaneously with the peptide mix in such a way that the dose for each peptide injected in mice was 5μg. After two weeks, a second injection of the same concentration was administered after emulsifying these peptides in incomplete Freund’s adjuvant. Three days later the frequencies of peptide specific CD8^+^ T cells were analyzed by tetramer staining of PBMCs. In separate experiments the draining LN cells from immunized animals were collected and expanded *in vitro* by stimulating with peptides (10μg/ml) in the presence of IL–2 (10ng/ml) for three days in a humidified CO_2_ incubator. The expanded cells were labeled with CFSE and a total of 5x10^6^ cells were adoptively transferred in IFNR KO mice. One day later the animals were infected with PPRV to measure the disease modifying activity as well as the virus–induced proliferation of transferred CD8^+^ T cells.

### Statistical Analysis

To measure the level of statistical significance, the data was subjected to analysis using ANOVA, Student t test or the Gehan–Breslow–Wilcoxon test as indicated in the figure legends. Graph Pad Prism v8.4.3 was used for such analysis. The level of significance was determined as P < 0.05 *, P < 0.01 **, P < 0.001***, P < 0.0001**** and ns for P > 0.05.

## Results

### Mice Genetically Ablated of IFNRs Are Susceptible to PPRV Infection

**Figure 1:**
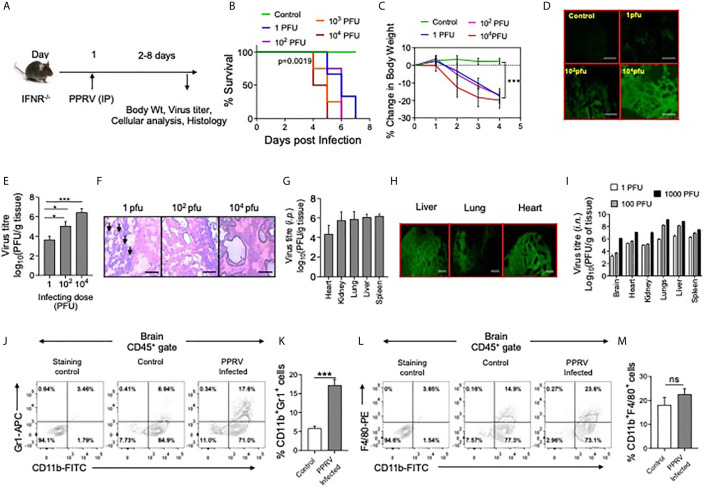
IFNR KO mice are susceptible to PPRV infection. **(A)** The schematic of experiments is shown. **(B)** IFNR KO mice (n=3 or 4 per group) were *i.p.* infected with indicated doses of PPRV and the survival analysis for these animals were performed. The level of statistical significance was determined by Gehan–Breslow–Wilcoxon test for survival analysis. **(C–H)** IFNR KO mice were *i.p*. infected with indicated doses of PPRV and different organs were collected at 4dpi to measure the presence of viral antigens and replicating virus in different organs. **(C)** Graphical representation of percentage change in body weight of IFNR KO mice after infection with different doses of PPRV from each group is shown. **(D)** Fluorescent microscopic images off brain tissues of the infected IFNR KO mice are shown as detected by anti–PPRV **(H)** mAbs. The images were captured at 100x magnification. **(E)** Bar diagrams show virus titers from brain tissues of PPRV infected IFNR KO mice administered with varying doses. Representative micrographs from H&E–stained samples of the brain tissue sections from PPRV infected animals are shown in **(F)**. **(H)** Bar diagrams show virus titers obtained from different organs of IFNR KO mice after intraperitoneal infection with PPRV. **(G)** Representative fluorescent microscopic images of different organs of virus infected IFNRKO mice stained with antibodies for PPRV antigens are shown. **(I)** Bar diagrams show virus titers calculated from different organs of IFNR KO mice (n=4/gp) infected using intra–nasal route with different doses of PPRV. **(B–I)** One–way ANOVA analysis was used and Tukey’s multiple comparisons test was used for determining the statistical significance values between groups and are represented as following, p < 0.05 *, p < 0.001 ***. **(J–M)** Innate immune cells were analyzed from brain tissues of PPRV infected (*i.p.*) IFNR KO mice. **(J**, **K)** Representative FACS plots show the CD11b^+^Gr1^+^ cells in control and PPRV infected mice and bar diagrams show the frequencies of such cells from the animals from different groups. **(L**, **M)** Representative FACS plots show the frequencies of CD11b^+^F4/80^+^ cells while bar diagrams show their frequencies in control and PPRV infected mice. The experiments were repeated more than two times. The data represented here is from one such experiments. Student t test were performed for determining the significance levels in control and infected groups. p < 0.05 *, p < 0.001 *** and ns for not significant.

We tested whether or not mice unable to mount IFN response because of genetic ablation of the receptors were susceptible to PPRV. Different doses of PPRV (1, 10^2^,10^3^ or 10^4^ PFU) were *i.p.* inoculated into IFNR KO mice and the disease progression was monitored up to eight dpi (**Figure 1A**). All the infected animals succumbed to the infection and their survival duration was influenced by the initial inoculum (**Figure 1B**). Accordingly, animals infected with the high dose of PPRV died earlier as compared to those infected with the lower dose (**Figure 1B**). We then analyzed other physiological parameters such as the alteration in body weights, behavioral changes and thermoregulatory effects in infected animals. All the infected animals gradually lost their body weights, developed encephalitic lesions and became hypothermic (**Figure 1C** and data not shown). By 7dpi, all the animals succumbed to the infection irrespective of virus inoculum size (**Figures 1B, C**). In similar experiments, PPRV infected WT mice remained refractory to the PPRV–induced disease as no clinical signs were evident even when a very high infecting dose (5x10^6^ PFU) was used (**Figures 1B, C**, **4B**). The results underscored the critical role played by IFN signaling in providing an early defense against PPRV infection in mice. Furthermore, the PPRV exposed innate immune cells (RAW macrophages and BMDMs) mounted a rapid type I and type II IFN response (**Figure S1**).

Since, PPRV–infected IFNR KO mice developed encephalitic lesions, we measured PPRV antigens in brain tissue sections prepared from such animals using anti–PPRV (H) monoclonal antibody by fluorescent microscopy (**Figure 1D**). PPRV antigens were abundant in brain tissue sections particularly at high doses of the virus inoculum (**Figure 1D**). Furthermore, a dose dependent increase in the replicating PPRV titers were found in the homogenized brain tissues (**Figure 1E**). Accordingly, the mean titers of replicating virus were 3.6 ± 0.3, 5.0 ± 0.4 and 6.4 ± 0.3 log_10_PFU/g of brain tissues at the infecting dose of 1, 10^2^ and 10^4^ PFU, respectively (**Figure 1E**). The results suggested for an efficient replication of virus in the brain tissues of PPRV infected IFNR KO mice. The IFNR KO mice were susceptible to PPRV infection at very low dose of 1PFU. We, therefore, investigated whether or not the infecting dose qualitatively influenced the disease phenotype. The brain tissue sections from animals infected with varying doses of PPRV were stained with H&E to analyze infiltrating inflammatory cells (**Figure 1F**). The swelling of meninges was observed in the brain sections from animals infected with a high dose of PPRV (10^4^ PFU) (**Figure 1F**, right panel). Interestingly, the stained sections of brain tissues from animals infected with a low dose of PPRV (1PFU) showed greater leukocytic infiltration as compared to those with high dose (10^4^PFU) (**Figure 1F**, left panel). We also investigated the presence of replicating PPRV in different organs of mice infected with 1PFU of the virus. We observed the viral titers of 4.3 ± 0.8, 5.8 ± 0.8, 6.0 ± 0.3, 6.1 ± 0.2 and 5.7 ± 0.8 log_10_PFU/g of tissue in heart, lungs, liver, spleen and kidneys, respectively at 6dpi (**Figure 1G**). With an increase of the virus inoculum size, more PPRV titers were obtained (data not shown). The viral antigens were abundantly present in different organs of PPRV infected IFNR KO mice as analyzed by fluorescent microscopy (**Figure 1H**). In another approach, we infected IFNR KO mice with PPRV (1,10^2^ and 10^4^ PFU) using intranasal route (*i.n*) and measured replicating virus in different organs. All the investigated organs showed high levels of replicating virus and the extent of virus loads increased with increasing amounts of the infecting virus dose (**Figure 1I**). Our results therefore suggested for an active replication of PPRV in different organs of IFNR KO mice irrespective of the inoculation route.

We also measured the phenotype of leukocytes (CD45^+^ cells) in single cell suspension prepared from brain tissues of PPRV infected mice by flow cytometry. Significantly higher frequencies of neutrophils (CD11b^+^Gr1^+^) were present in the brain samples of PPRV infected mice as compared to those from uninfected controls (**Figures 1J, K**). The frequencies of macrophages (CD11b^+^F4/80^+^) were also higher but the results were not statistically different between control and infected mice (**Figures 1L, M**). The increased frequencies of innate immune cells further supported our data on phenotypic lesions involving CNS and the development of encephalitis in PPRV infected IFNR KO mice.

### Innate Immune Cells Are Permissive to PPRV Infectivity

**Figure 2:**
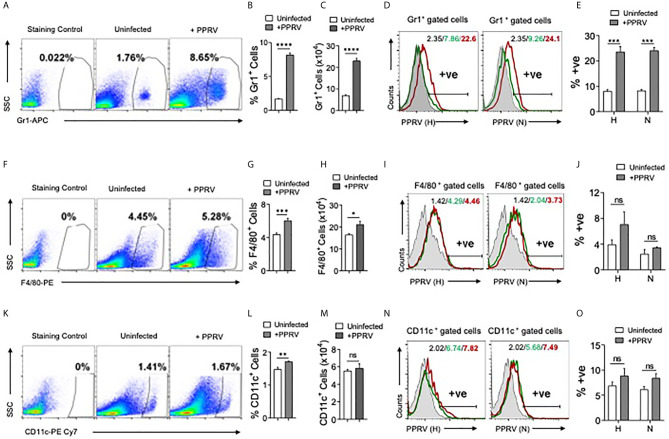
Determining PPRV infectivity of innate immune cells in IFNR KO mice. IFNR KO mice (n=4/gp) were *i.p* infected with 1PFU of PPRV. The expansion of innate immune cells and the presence of viral antigens in these cells were measured in spleen. **(A)** Representative FACS plots show the frequencies of Gr1^+^ cells among live cell gate in uninfected and PPRV infected mice. **(B**, **C)** Bar diagrams show cumulative data for the frequencies and counts of Gr1^+^ cells in uninfected and PPRV infected mice from one of the representative experiments. **(D**, **E)** Intracellular staining was performed to measure the presence of PPRV antigens in Gr1^+^ cells using anti–PPRV (H) and anti–PPRV ****(N)**** monoclonal antibodies. **(D)** Representative overlaid histograms show the frequency of PPRV (H, N)^+^ Gr1^+^ cells. **(E)** Bar diagrams show cumulative data as percent positive Gr1^+^PPRV (H)^+^ and PPRV (N)^+^ cells from one such experiment. **(F)** Representative FACS plots show the frequencies of F4/80^+^ cells among live cell gate in uninfected and PPRV infected mice. **(G**, **H)** Bar diagrams show cumulative data for the frequencies and numbers of F4/80^+^ cells in uninfected and PPRV infected mice. **(I**, **J)** Intracellular staining was performed to measure the presence of PPRV antigens in F4/80^+^ cells using anti–PPRV (H) and anti–PPRV (N) monoclonal antibodies. **(I)** Representative overlaid histograms show the frequency of PPRV (H, N)^+^ F4/80^+^ cells from one such experiment. **(J)** Bar diagrams show the percentage of F4/80^+^ cells expressing PPRV (H) and PPRV (N) proteins. **(K)** Representative FACS plots show the frequencies of CD11c^+^ cells among live cell gate in uninfected and PPRV infected mice. **(L**, **M)** Bar diagrams show cumulative data for the frequencies and numbers of CD11c^+^ cells in uninfected and PPRV infected mice. **(N, O)** Intracellular staining was performed to measure the presence of PPRV antigens in CD11c^+^ cells using anti–PPRV (H) and anti–PPRV (N) monoclonal antibodies. N. Representative overlaid histograms show the percentage of PPRV (H and N)^+^ CD11c^+^ cells. **(O)** Bar diagrams show the percentage of CD11c^+^ cells expressing PPRV (H) and PPRV (N) proteins. The coloring in the overlaid histograms indicate as shaded region– control, green line– uninfected and red line– PPRV infected mice. The experiments were repeated two times. The bar diagrams are represented as mean ± SEM. Different groups were analyzed by unpaired students t–test. Statistical significance values are represented as following, p < 0.05 *, p < 0.01 **, p < 0.001 ***, p < 0.0001 **** and ns for not significant.

PPRV infected IFNR KO animals infected with low doses (1PFU) of the virus showed infiltration of innate immune cells in the brain tissues. We then analyzed immune response in spleens of such mice (**Figure 2**). The frequencies and counts of innate immune cells such as neutrophils (CD11b^+^Gr1^+^) increased by up to 5–fold in the infected mice as compared to their uninfected counterparts (**Figures 2A–C**). The proportion of macrophages (CD11b^+^F4/80^+^) and myeloid dendritic cells (mDCs, CD11b^+^CD11c^+^) also increased significantly in the infected mice as compared to uninfected controls but these cells were less abundant than neutrophils (**Figures 2F–H, K–M**).

A rapid induction of innate immune cells after PPRV infection and the expression of encephalitic lesions in infected IFNR KO mice even at low dose of the virus led us to explore the possibility of virus transport to CNS by such cells. Therefore, we measured the viral invasion of innate immune cells using intracellular staining for the viral proteins. Neutrophils (Gr1^+^), macrophages (F4/80^+^), and DCs (CD11c^+^) from uninfected and PPRV infected animals were analyzed by immunostaining for the presence of PPRV hemagglutinin (H) and nucleocapsid (N) proteins (24). In morbilliviruses, N proteins are highly conserved and constitute a major component of ribonucleoprotein complex while H proteins help in the virus attachment to target cells. A significantly higher frequencies of neutrophils isolated from PPRV infected mice scored positive for H and N viral proteins (**Figures 2D, E**). PPRV proteins were also detectable in the isolated macrophages and DCs of infected animals but the results were statistically insignificant between control and PPRV infected animals (**Figures 2I, J, N, O**). Therefore, neutrophils and perhaps other innate immune cells by virtue of their PPRV infectivity could transport the virus to different tissues.

### Innate Immune Cells Transport PPRV to Central Nervous System

**Figure 3:**
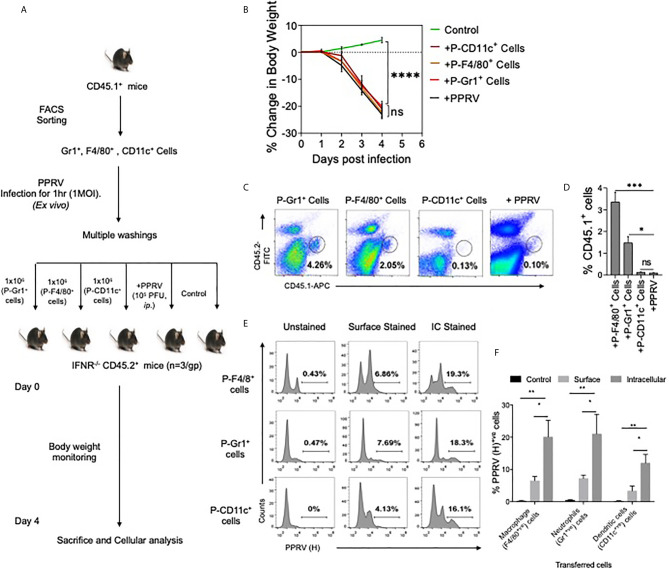
Innate immune cells serve as the carrier of PPRV to brain tissues. **(A)** Flow–chart shows the experimental design. FACS sorted neutrophils, macrophages and dendritic cells from B6 CD45.1/J mice (CD45.1^+^) were pulsed with PPRV (1MOI) and transferred into CD45.2^+^ IFNR KO mice. One group of mice did not receive any cells but were infected with PPRV (10^5^PFU). At 4dpi, brain tissues were analyzed for CD45.1 and CD45.2 cells. **(B)** Graphical representation of the percent change in body weight in the IFNR KO mice in only the infected group and recipient mice. **(C)** Representative FACS plots shows the presence of CD45.1^+^ cells and CD45.2^+^ cells in the brain tissues and bar diagrams **(D)** show the cumulative frequencies of expanded donor cells recovered from brain suspensions from animals in one such experiment. **(E)** FACS plots show the presence of viral antigens in F4/80^+^, CD11c^+^ and GR1^+^ cells after multiple washings. **(F)** Bar diagrams represent the frequencies for the presence of PPRV viral antigens in different cells. For each group of recipients three animals were used and the experiments were performed two times. ANOVA test was performed for determining the statistical significance values and are represented as following, p < 0.05 *, p < 0.01**, p < 0.001 ***, p < 0.0001**** and ns for not significant.

We then investigated whether or not innate immune cells helped transport PPRV to CNS. PPRV infected innate immune cells from CD45.1^+^ congenic mice were adoptively transferred in IFNR KO mice and the disease progression was monitored in recipients (**Figure 3A**). Neutrophils (Gr1^+^ cells), macrophages (F4/80^+^ cells) and DCs (CD11c^+^ cells) were FACS sorted from CD45.1^+^ congenic mice (CD45.1^+^/CD45.2^–^). The sorted cells were infected *ex vivo* with PPRV (1MOI) and washed extensively thereafter. These cells were then transferred into sex matched IFNR KO mice (CD45.2^+^/CD45.1^–^) (**Figure 3A**). Animals in one group did not receive any cells but were infected with cell–free PPRV. The transferred PPRV–pulsed cells resulted in a patent infection in recipients and the progression of disease occurred at similar levels as was observed in PPRV infected animals (**Figure 3B**). We also recovered elevated frequencies of donor neutrophils (CD45.1^+^CD11b^+^Gr1^+^, ~ 4% of live cells) and donor macrophages (CD45.1^+^CD11b^+^F4/80^+^, ~ 2% of live cells) from the brain tissues of recipient mice as compared to the background levels (<0.1% of live cells), where animals were directly infected with PPRV (**Figures 3C, D**). The data suggested that infected innate immune cells particularly the neutrophils and macrophages could support PPRV replication and transport the virus to different organs. To further investigate whether or not PPRV can be internalized by the virus–pulsed cells, we measured viral antigens in such cells by performing both surface and intracellular staining with anti–PPRV (H) antibody and analyzing such cells by flow cytometry (**Figures 3E, F**). Three to four–fold higher frequencies of cells were positive for intracellular PPRV antigens as compared to those that scored positive for surface antigens of the virus (**Figure 3F**). These results indicated that the virus could either be internalized by innate immune cells or remain surface associated and such cells could transport PPRV to distant sites such as the CNS.

### PPRV Induces Lung Pathologies in IFNR KO Animals Infected *via* Intranasal Route

**Figure 4:**
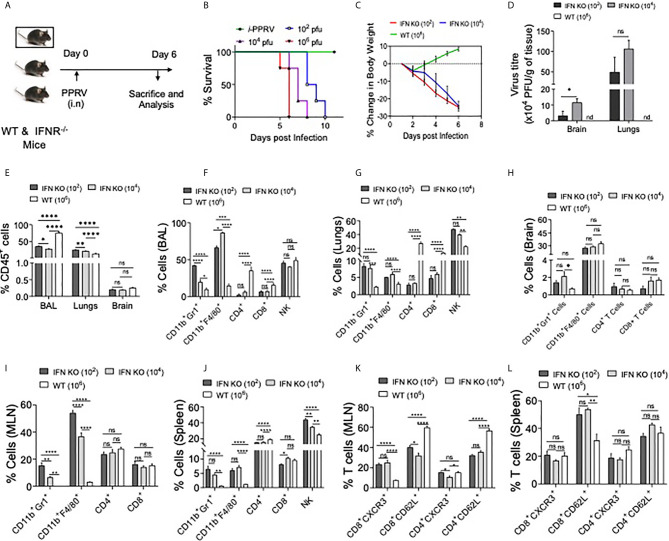
IFNR KO mice infected with PPRV develop lung pathologies and mount innate as well as adaptive immune responses. **(A)** Schematic shows IFNR KO (n=5 and WT C57BL/6 mice (n=5) infected with indicated doses of PPRV *via* intranasal route and animals were analyzed for their survival and percent change in body weights **(B**, **C)**. The level of statistical significance in change in body weight was determined by two–way ANOVA analysis. Terminally anaesthetized animals were analyzed for measuring the cellular infiltration in BAL, lungs and lymphoid organs at 7dpi. **(D)** Bar diagrams show the virus titers calculated from brain tissues and lungs of PPRV infected animals at indicated doses. **(E)** The frequencies of leukocytes (CD45^+^ cells) obtained from BAL, lungs and brain tissues of infected WT and IFNR KO mice are shown. **(F**, **G)** Bar diagrams show the frequencies of CD11b^+^Gr1^+^ cells, CD11b^+^F4/80^+^ Cells, CD4^+^ T cells, CD8^+^ T cells and NK1.1^+^ cells in BAL and lungs of infected animals among the gated CD45^+^ cells. **(H**, **I)** Bar diagrams show the frequencies of CD11b^+^Gr1^+^ cells, CD11b^+^F4/80^+^ Cells, CD4^+^ T cells and CD8^+^ T cells in mediastinal lymph node and brain of infected animals as percent positive. **(J)** Bar diagrams show the frequencies of CD11b^+^Gr1^+^ cells, CD11b^+^F4/80^+^ Cells, CD4^+^ T cells, CD8^+^ T cells and NK1.1^+^ cells as percent positive from spleen samples. **(K**, **L)** Bar diagrams show the frequencies of CD8^+^CXCR3^+^ T cells, CD8^+^CD62L^+^ T cells, CD4^+^CXCR3^+^ T cells and CD4^+^CD62L^+^ T cells in mediastinal lymph nodes and spleen, respectively. The experiments were repeated three times. The data was analyzed by two–way ANOVA for determining the statistical significance values. The levels of significance are represented as following, p < 0.05 *, p < 0.01 **, p < 0.001 ***, p < 0.0001 **** and ns for not significant.

PPRV causes respiratory disease in infected small ruminants and the infection spreads among animals in the herd due to their closer association, we therefore investigated its pathogenesis in mice infected *via i.n* route. IFNR KO animals were infected with varying dose of PPRV and were analyzed for their survival, body weights and other physiological parameters (**Figure 4**). The survival analysis of animals showed a significant effect of inoculum sizes but all the animals eventually succumbed to the infection (**Figure 4B**). The animals infected with the lower dose (10^2^ PFU) succumbed to the infection later than those infected with the higher doses (10^6^ PFU). For performing cellular analysis in the infected animals both at tissue sites and lymphoid organs, additional groups of infected animals were sacrificed at a time when either group lost more than 20% of their body weights (**Figure 4A** and **Figures S2**-**S5**). WT animals were infected with a high dose (10^6^ PFU/mouse) as all of the WT animals that were *i.p* infected animals with higher dose of PPRV survived (**Figures 1B, C**, data not shown). Infected WT animals reduced their body weights only transiently followed by a rapid recovery until the termination of experiments while the infected IFNR KO animals reduced their body weights at both doses (10^2^ PFU and 10^4^ PFU) of PPRV until the termination of experiments (**Figure 4C**). The infected animals were euthanized at 6dpi to perform cellular analysis in bronchoalveolar lavage (BAL) fluids, lungs, brain and lymphoid organs were collected. The viral load in lungs and brain tissues were also measured by plaque assays. The replicating virus particles were abundant in lungs and brain tissue of the infected animals and more of the viral particles were recovered from animals receiving high dose of the virus (**Figure 4D**). More virus particles were detected in lungs than in brain tissues at both the infecting doses while no detectable virus particles were present in the brain as well as the lung tissues of infected WT mice (**Figure 4D**). To assess the inflammatory response of different organs, we measured leukocytic infiltration and observed an approximately 1.5–fold higher frequencies of such cells (CD45^+^ cells) in the BALs of WT animals as compared to the IFNR KO animals. We observed lower frequencies of CD45^+^ cells in lungs of PPRV infected WT mice as compared to the IFNR KO mice indicating a lesser extent of inflammation in the lung tissues of WT animals (**Figure 4E**). This could suggest that an efficient viral control was achieved in WT mice that prevented the establishment of a patent lung infection. We also observed an enhanced infiltration of CD45^+^ cells in BAL (~ 42% vs 30%) as well as lung tissues (27% vs 19%) of IFNR KO animals infected with the low (10^2^ PFU) and high (10^4^ PFU) dose of PPRV, respectively (**Figure 4E**). These results indicated immunopathological lesion at lower infecting dose. Both WT and IFN RKO mice showed minimal infiltration of leukocytes in brain tissues (**Figure 4E**). We then phenotypically characterized different immune cells among the leukocyte populations from different organs to gain insights into their potentially discordant migratory behaviors observed in *i.p* and *i.n* route of infection. The relative abundance of macrophages (CD11b^+^F4/80^+^), neutrophils (CD11b^+^Gr1^+^), NK cells (NK1.1^+^ cells), helper (CD4^+^) and cytotoxic (CD8^+^) T cells was measured in the non–lymphoid as well as lymphoid organs of infected WT and IFNR KO mice (**Figures 4F–J**). Up to five–fold reduced frequencies of macrophages and neutrophils were found in BAL, lung tissues, brain, mediastinal LNs and spleens of infected WT animals as compared to IFNR KO mice (**Figures 4F–J**, respectively). The frequencies of NK cells were similar in BAL but decreased in the lung and spleen of the PPRV infected WT animals as compared to those in IFNR KO mice (**Figures 4F, G, J**). The frequencies of both CD4^+^ and CD8^+^ T cells in BAL and lung tissues increased up to 40–fold in PPRV infected WT mice in comparison to those in the IFNR KO mice (**Figures 4F, G**). The enhanced frequencies of NK cells but reduced frequencies of neutrophils and macrophages were observed in BAL and lung tissues of WT mice (**Figures 4F, G**). These results indicated that an early induction of T cell response along with the activity of NK cells could provide an efficient control of PPRV in WT animals. With an efficient viral control at infection sites, the animals did not develop pathological lesions and recovered rapidly.

BAL and mediastinal LNs of infected IFNR KO animals infected with different doses of the virus exhibited two to three–fold higher frequencies of neutrophils at the lower infecting dose as compared to the high dose (**Figures 4F, I**). However, such an increase was not observed in lung tissues, brain and spleen samples of the infected animals (**Figures 4G, H, J**). The inverse correlation with PPRV inoculum size and the recruitment of macrophages was also not evident in lungs, brain and spleens of IFNR KO animals infected with PPRV (**Figures 4F, G, I**). These results could suggest for aberrant activation and recruitment of different immune cells to different tissue and such a phenomenon might depend on the dose of infecting virus.

We further analyzed the activation profile of T cells in draining mediastinal LNs and spleens of PPRV infected animals. A significant proportion of CD4^+^ T cells displayed an activation profile (CD62L^lo^ or CXCR3^+^) in LN and spleen of WT animals but the frequencies of such cells were up to three–fold higher in the IFNR KO animals (**Figures 4K, L** and **Figure S4**). These results could mean that the activated T cells in WT animals helped control the virus owing to their migration at tissue sites and those in IFNR KO mice failed to do so. We also observed a distinct population of CD62L^hi^CXCR3^+^CD8^+^ T cells that were approximately six and three–fold more abundant in mediastinal LN and spleens, respectively, of the infected WT animals as compared to those in IFNR KO mice (**Figure S4**). Such cells were shown to have potent functions (25, 26). However, their further role in PPRV pathogenesis was not investigated.

Taken together, the analysis of immune cells suggested for the pathological role of neutrophils in PPRV infection while NK cells and T cells could be protective. Accordingly, such responses were clearly evident in WT animals that efficiently controlled the infection.

### IFN Responsive CD8^+^ T Cells Delays Mortality in PPRV Infected IFNR KO Mice

**Figure 5:**
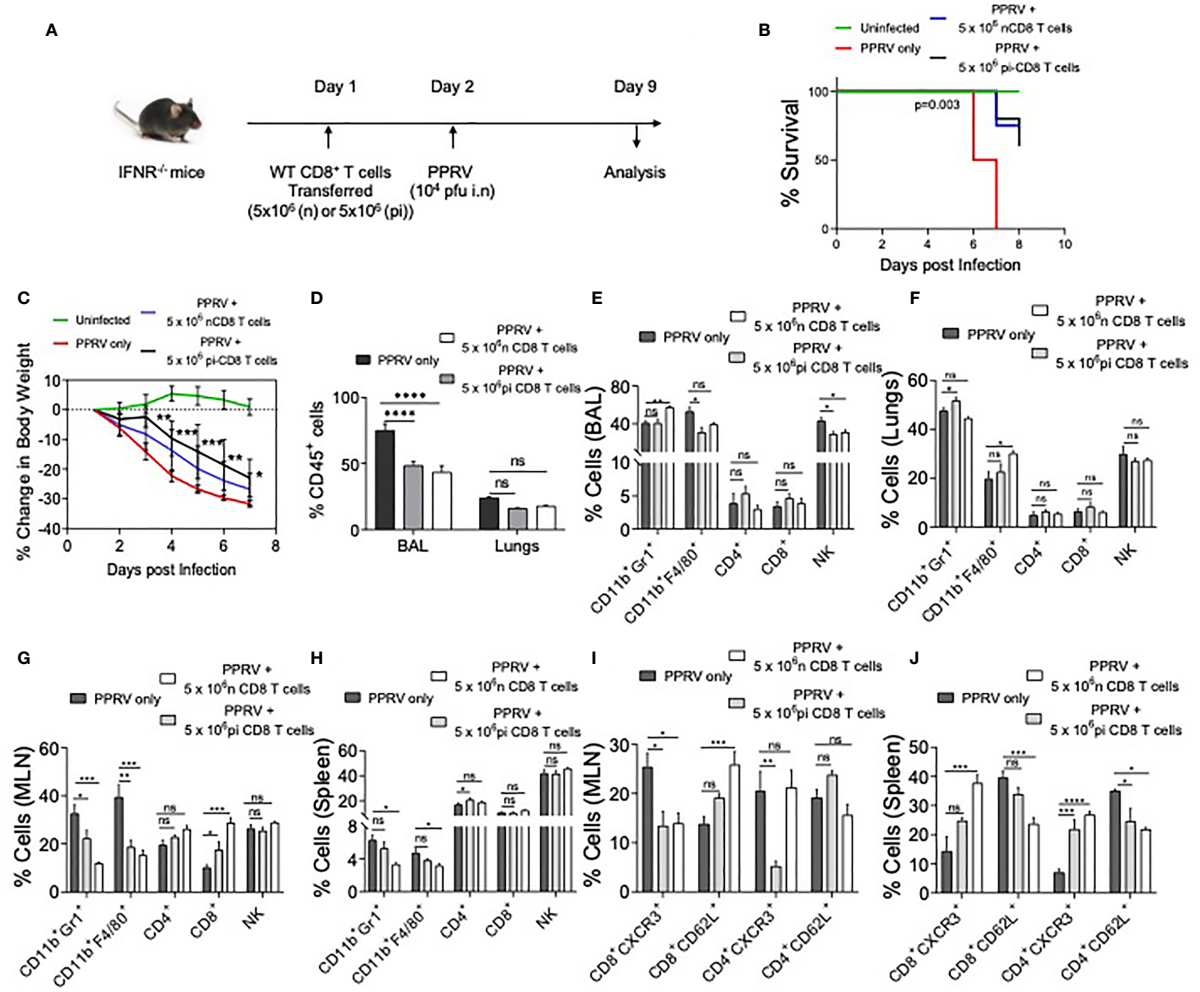
WT CD8^+^ T cells delay mortality in PPRV infected IFNR KO mice. A schematic of the experiments is shown. 5x10^6^ of CD8^+^ T cells from naïve (n=6) or CD8^+^ T cells from previously PPRV infected WT mice (n=6) collected five days later were transferred into IFNR KO mice. The control group (n=4) did not receive any cells. Next day, the recipient animals were infected with PPRV (10^4^ PFU) intranasally. The disease progression, survival and cellular analysis of immune cells were performed. **(B)** The survival in different groups of animals is shown. The results were analyzed by Gehan–Breslow–Wilcoxon test. **(C)** Percent change in body weight of mice from each group is shown. The level of statistical significance was determined by two–way ANOVA analysis. **(D)** The frequencies of leukocytes (CD45^+^ cells) in BAL and lungs of infected mice are shown by bar diagrams. **(E–H)** The frequencies of macrophages (CD11b^+^F4/80^+^), neutrophils (CD11b^+^Gr1^+^), NK cells (NK1.1^+^) and T cells (both CD4^+^ and CD8^+^ T cells) are shown in the BAL **(E)**, lungs **(F)**, mediastinal lymph node **(G)** and spleen **(H)** of infected animals by bar diagrams. **(I)** and **(J)** The phenotypic characterization CD4^+^ T cells and CD8^+^ T cells for the indicated markers were performed. The percent positive cells frequencies for the indicated markers are shown for mediastinal lymph node **(I)** and spleen **(J)** of infected animals by bar diagrams. The experiments were performed three times. The data was analyzed by 2–way ANOVA using Tukey’s multiple comparisons test for determining the statistical significance values and are represented as following, p < 0.05 *, p < 0.01 **, p < 0.001 ***, p < 0.0001 **** and ns for not significant.

We established the susceptibility of IFNR KO mice to PPRV, the potential spread of PPRV by infected innate immune cells and expansion of innate immune cells as well as T cells in PPRV infected WT mice. We then explored whether WT T cells could be protective in PPRV infected IFNR KO animals. We transferred WT CD8^+^ T cells from WT mice previously infected with PPRV or from naïve animals to measure their ability to confer protection to PPRV infected IFNR KO mice. We transferred 5x10^6^ of purified WT CD8^+^ T cells collected from naïve or PPRV infected mice into IFNR KO mice. The recipients were then infected with PPRV (10^4^PFU) (**Figure 5A**). The survival analysis showed a significant advantage conferred to the infected IFNR KO mice by such cells in delaying mortality (**Figure 5B**). The separate groups of PPRV infected mice transferred with CD8^+^ T cells were analyzed for a change in their body weights and cellular analysis on 7dpi (**Figures S7** and **S8**). CD8^+^ T cell recipient mice showed significantly less reduction in their body weight by 6dpi (**Figure 5C**). Cellular analysis showed that ~70% of CD45^+^ cells were present in the BAL of PPRV infected animals and their frequencies decreased to 40% in CD8^+^ T cells recipients (**Figure 5D**). Similarly, the frequencies of CD45^+^ cells reduced from 25% to 15% in the lung tissues of infected animals that received CD8^+^ T cells (**Figure 5D**). Neutrophil levels increased in the BAL of CD8^+^ T cell recipient IFNR KO mice by ~1.5 fold but the macrophages and NK cells decreased by ~ 10% (**Figure 5E**). In lung tissues, a slight increase in the cellular infiltrates was observed in recipients of CD8^+^ T cells (**Figure 5F**). A reduction in the frequencies of innate immune cells such as neutrophils and macrophages but increase in those of CD4^+^ and CD8^+^ T cells were recorded in the MLN and spleens of infected animals that received WT CD8^+^ T cells (**Figures 5G, H**). A lower frequency of CD4^+^ and CD8^+^ T cells were CXCR3 positive while more of such cells expressed CD62L in the draining mediastinal lymph node of the CD8^+^ T cell recipients IFNR KO mice infected with PPRV as compared to the control animals (**Figure 5I**). These results might indicate that CD8^+^ T cells were retained in the LNs of infected animals probably due to chemokines build up and a less efficient gradient generation that could have hampered their exit. Such a situation could contribute to an inefficient viral control. In the spleen of WT CD8^+^ T cell recipients as compared to control mice, however, more of CD4^+^ and CD8^+^ T cells expressed CXCR3 but the frequencies of CD62L positive cells reduced (**Figure 5J**). While we observed protective roles of transferred WT CD8^+^ T cells in infected IFNR KO mice, the mechanism were less well defined.

In additional experiments, we transferred FACS sorted CD4^+^ and CD8^+^ T cells from WT mice into IFNR KO animals. As the immune cells of adaptive branch were less frequent in IFNR KO mice as compared to the WT animals, we considered allowing the homeostatic expansion of donor cells for more than a month (40 days) following which the animals were infected with PPRV (**Figure S6A**). In comparison to infected controls that gradually lost their body weights, a significantly lesser reduction in body weights was observed in CD8^+^ T cell recipients (**Figure S6B**). WT CD4^+^ T cell recipient mice and those receiving bone marrow cells did not show alteration in the rates or the kinetics of body weight loss (**Figure S6B**, data not shown). The activation status of CD8^+^ and CD4^+^ T cells of control and T cell recipient mice revealed higher frequencies of CD4^+^ and CD8^+^ T cells that expressed high levels of CD44 only in the recipients of WT CD8^+^ T cells (**Figures S6C–F**, data not shown). These results suggested for the protective anti–viral roles of WT CD8^+^ T cells in this model.

At present a detailed analysis in a seemingly dichotomous activation profile of T cells subset is lacking. However, the aberrant activation profile of T cells in PPRV infected IFNR KO mice as well as a crucial role of functionally competent CD8^+^ T cells in mitigating PPRV pathogenesis were evident.

### Identification of Immunogenic CD8^+^ T Cell Epitopes of PPRV *In Silico*

**Figure 6:**
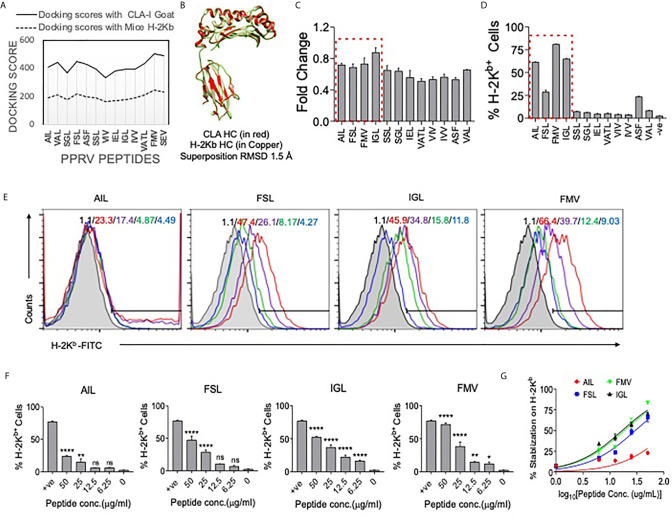
Molecular docking of predicted peptides binding with H–2K^b^ and CLA–I and their immunogenicity. High scoring peptides and their origin of PPRV protein were selected and individual peptides were separately docked with H–2K^b^ and goat class I MHC (CLA–1). **(A)** A comparative analysis of docking scores of H–2K^b^ and CLA–I for the predicted PPRV peptides is shown. **(B)** H–2K^b^ and CLA–1 were superimposed at RMSD value of 1.5A. **(C)** An ELISA was performed for measuring the stabilization of H–2K^b^ monomers by synthetic peptides predicted to be immunogenic. The bar diagrams show the fold change values for the respective peptides as compared to the control peptide (SIINFEKL). **(D)** Representative bar diagrams shows the surface MHC I stabilization by the selected peptides in RMA/S cell lines. **(E)** Representative overlay histograms for different concentrations (50μg/ml, 25μg/ml, 12.5μg/ml and 6.25μg/ml) of indicated peptides show their ability to stabilize surface MHC I molecules. The values represent the percent positive cells at different concentrations of peptides. The color indications for the overlaid histograms indicate as follow: shaded region– control, red line– 50μg/ml, violet line– 25μg/ml, green line–12.5μg/ml and blue line–6.25μg/ml conc. of the indicated peptide. **(F)** Bar diagrams show percent H–2K^b^ positive cells for different concentrations of the indicated peptides. **(G)** Line graph shows log EC_50_ values for different peptides for stabilizing MHC I in pulsed RMA/s cells. One–way ANOVA were performed for determining the statistical significance values and are represented as following, p < 0.05 *, p < 0.01 **, p < 0.001 **** and ns for not significant.

We identified immunogenic epitopes of PPRV that could induce specific CD8^+^ T cells response in mice. Initially, all the six PPRV proteins were analyzed for peptide selection (**Table S2**). The proteins such as hemagglutinin, matrix, nucleocapsid, phosphoprotein, large polymerase and fusion proteins of PPRV are required for viral fusion to cell membrane or to the receptor attachment as well as in the replication process. However, we focused our subsequent analysis on four PPRV proteins critically involved in the viral pathogenesis and if the discovered immunogenic epitopes are incorporated in the formulation of a candidate vaccine disease progression could be modulated. The proteins were then analyzed for predicting H–2K^b^ restricted epitopes using IEDB (**Tables S3**, **S4**). We focused our analysis on the structural proteins because such proteins are critical in virus assembly and envelope formation (24). Peptides with low percentile ranks and the IC_50_ value of <200nM were selected. Out of the list generated, top 12 ranked peptides (three from each protein) were synthesized for further analysis (**Tables S3**, **S4**). The peptides included in analysis were IVVRRTAGV, VAFNILVTL, FMYLFLLGV (matrix protein), FSAGAYPLL, ASFILTIKF, SSITTRSRL (Nucleocapsid protein), VILDRERLV, IEHIFESPL, IGLVRDFGL (hemagglutinin protein) and (AILTFLFLL, VAILTFLFL, SGGDFLAIL (fusion protein). The peptides were subjected to *in silico* analysis by molecular docking as this represents one of the frequently used methods to predict the conformation of small–molecule ligands (27, 28). Docking of selected peptides from different proteins of PPRV against H–2K^b^ was performed and the models were analyzed using Chimera tool. The energy parameters of the best fitting structures among PPRV peptides were determined. The best docking results were obtained for PPRV peptides, FMYLFLLGV and FSAGAYPLL with H–2K^b^. FMYLFLLGV peptide even yielded better docking scores than the reference peptide FAPGNYPAL (SEV–9) of Sendai E virus used for H–2K^b^ (**Figure 6A**, **Figure S9**, and **Tables S3**, **S4**). Further analyses showed phenylalanine residues at position 1 and 5 in FMYLFLLGV peptide serving as the probable anchors. Similarly, for FSAGAYPLL peptide phenylalanine at position 1 and proline at position 7 were predicted to be the most probable anchors. Docking of peptides was also performed with class I MHC molecule of goat (CLA1) (**Figure 6A**, **Figure S9**, and **Table S4**). CLA–I and H–2K^b^ superposed near perfectly at an RMSD value of 1.5A in the molecular docking analysis (**Figure 6B**). The predicted peptides showed a similar trend of docking with CLA–1 molecule. The anchor residues as well as the docking scores of FMYLFLLGV and FSAGAYPLL peptides scored better in these analyses (**Figure 6B** and **Figure S9**). Interestingly, all the epitopes displayed better docking with CLA–1 than with H–2K^b^ indicating their immunogenicity in generating anti–viral response against PPRV infection in the natural host, goats. Therefore, a total of 12 peptides were predicted that could potentially be immunogenic in mice as well as in the natural host of PPRV goats.

### Assessing Class I MHC Stabilizing Potential of Predicted PPRV Epitopes Using Acellular and Cellular Assays

We tested high ranked peptides of PPRV for their class I MHC stabilization using acellular and cellular assays. SIINFEKL, an Ova derived peptide with known immunogenicity for H–2K^b^, was used as a positive control. We first measured the MHC stabilizing potential of these peptides by ELISA (21). The fold change values for each of the PPRV derivative peptides as compared to those for SIINFEKL peptide are shown in **Figure 6C**. The PPRV peptides, FSAGAYPLL, IGLVRDFGL, AILTFLFLL, SSITTRSRL, IVVRRTAGV, IEHIFESPL, VAFNILVTL, and VILDRERLV displayed higher values as compared to other peptides (**Figure 6C**).

The peptides were also analyzed for their class I MHC stabilizing activities using transporter associated with antigen processing and presentation (TAP) deficient RMA/S cells that express fewer class I MHC molecules on surface. The exogenously added immunogenic peptides could help stabilize the surface expression of class I MHC (29). Different PPRV peptides were added to serum starved RMA/S cells and the expression of MHC class I stabilization was measured by flow cytometry (**Figures 6D–G**). Out of the 12 peptides, four peptides derived from matrix protein (FMYLFLLGV), nucleocapsid protein (FSAGAYPLL), hemagglutinin protein (IGLVRDFGL) and the fusion protein (AILTFLFLL) of PPRV stabilized surface H–2K^b^ at greater levels than the other peptides (**Figures 6D–F**). Similar results were obtained when the mean fluorescence intensity (MFI) values were measured for the expression of H–2K^b^ by each of the peptides (data not shown). The log EC_50_ values for AILTFLFLL, FSAGAYPLL, IGLVRDFGL and FMYLFLLGV peptides were 2.098, 1.469, 1.228 and 1.268 μg/ml, respectively (**Figure 6G**). Using acellular and cellular assays, we identified four out of 12 high ranked PPRV–peptides to be immunogenic due to their ability to efficiently interact with H–2K^b^.

### Determining the Immunogenicity of PPRV Peptides in PPRV Infected or Immunized Mice

**Figure 7:**
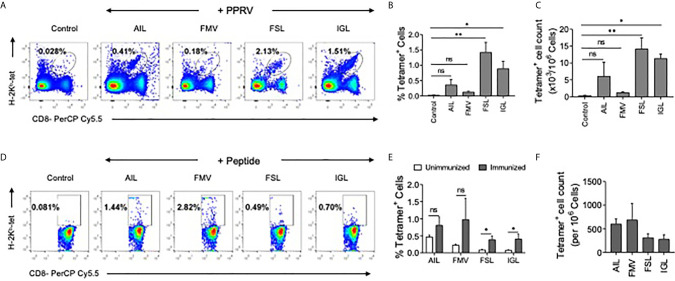
PPRV infected and PPRV–peptide immunized WT C57BL/6 mice expand virus–specific CD8^+^ T cells. **(A–C)** WT C57BL/6 mice (n=5) were *i.p*. infected with 5x10^6^ PFU of PPRV at an interval of 7 days. The analysis of the expanded cells was performed three days later by measuring the frequencies PPRV–specific CD8^+^ T cells by staining with arrays of tetramers. **(A)** Representative FACS plots from one such experiment shows the frequencies of class I MHC tetramer positive cells for the indicated peptides. **(B)** Frequencies of PPRV–specific CD8^+^ T cells are shown by bar diagrams. **(C)** The number of cells/million of PBMCs is shown by bar diagrams. **(D, E)** C57BL/6 mice (n=4) were immunized either with the cocktail of peptide (AILTFLFLL, FMYLFLLGV, FSAGAYPLL and IGLVRDFGL) each with 5μg/mouse in complete Freund’s adjuvant *via* subcutaneous route. After two weeks, a second injection of the same concentration was administered as an emulsion with incomplete Freund’s adjuvant. Three days later the frequencies of peptide specific CD8^+^ T cells were measured by tetramer staining of PBMCs. **(D)** Representative FACS plots show the frequencies of PPRV–specific CD8^+^ T cells. **(E**, **F)** Representative bar diagrams for the frequencies **(E)** and the number of cells/million of PBMCs is shown. The experiments were repeated two times and the statistical significance was measured by Student t test, p < 0.05 *, p < 0.01 ** and ns for not significant.

Whether or not the predicted PPRV peptides could stimulate and expand the virus–specific CD8^+^ T cells was analyzed in PPRV infected as well as immunized mice. WT C57BL/6 mice were refractory to PPRV infection, we therefore used a high dose of PPRV (5x10^6^ PFU) for infection. After 7 days, a boosting dose of similar magnitude was given. A significantly higher frequencies and numbers of H–2K^b^–p(PPRV)–tetramer positive CD8^+^ T cells reacted with AILTFLFLL, FSAGAYPLL and IGLVRDFGL peptides of PPRV (**Figures 7A–C**). However, the magnitudes of FMYLFLLGV peptides specific cells were not significantly different from the control animals (**Figures 7A–C**). This could be attributed to inefficient generation of class I tetramers with the FMYLFLLGV peptide. We also measured PPRV peptide specific CD8^+^ T cell responses in animals injected with the cocktail of four peptides (**Figures 7D–F**). The magnitudes of tetramer positive CD8^+^ T cell were higher for FSAGAYPLL, IGLVRDFGL peptides as compared to the other PPRV peptides in animals immunized with the cocktail of four peptides (**Figures 7D–F**). Our results showed that PPRV infected as well as the peptide immunized WT mice expanded PPRV–specific CD8^+^ T cells.

**Figure 8:**
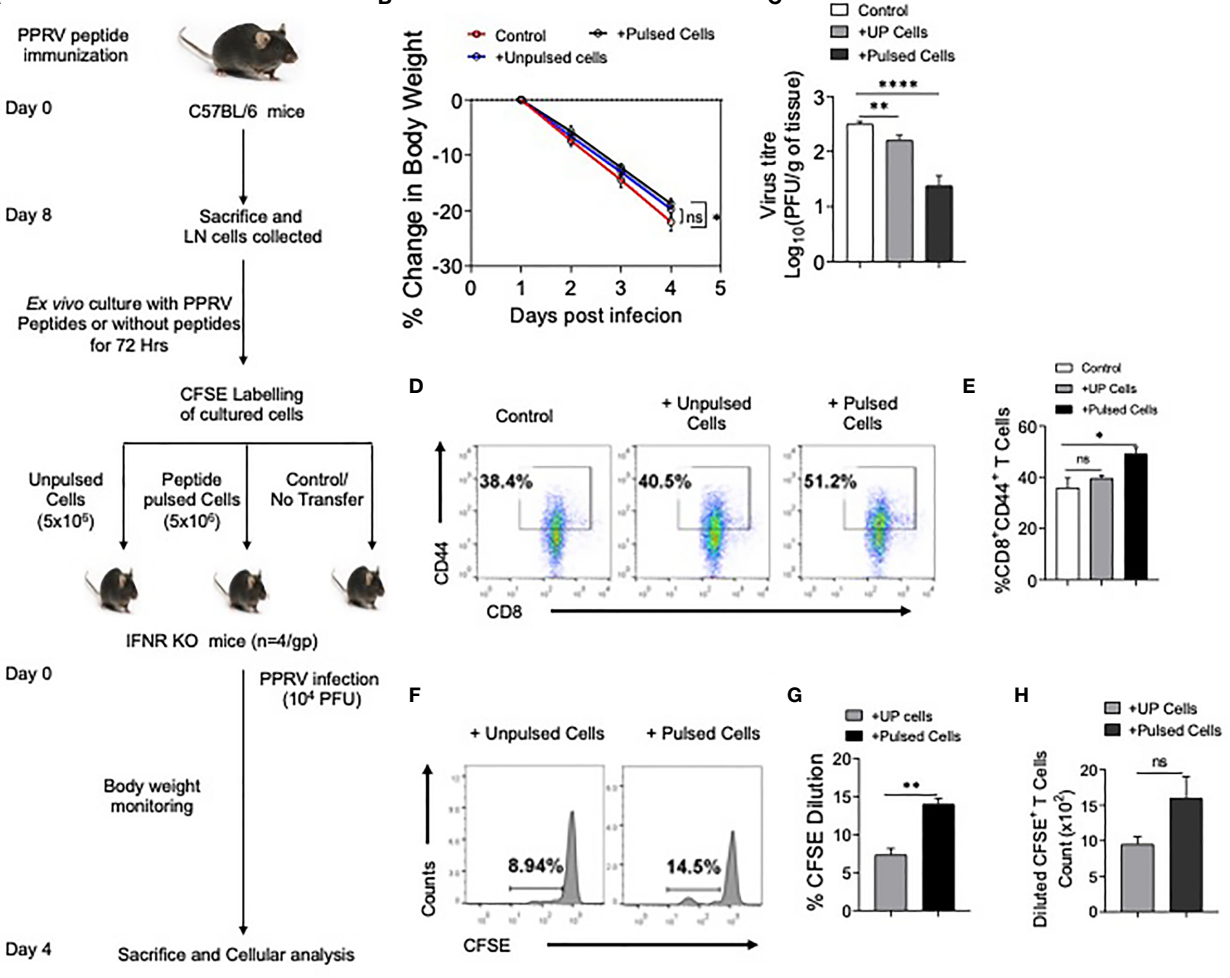
CD8^+^ T cells from peptide immunized WT mice responded in IFNR KO mice upon PPRV infection. **(A)** Experimental schematic is shown for immunization of WT mice (sub–cutaneous, shoulder region below neck) with emulsified cocktail of PPRV peptides (5μg/peptide/mouse). Eight days post immunization, axillary and brachial LNs as well as inguinal LN cells (Draining LNs) were collected and *in vitro* stimulated with 10ug/ml of each peptides in the presence of IL–2 (10ng/ml). The expanded cells were washed with PBS, labeled with CFSE and were adoptively transferred in IFNR KO mice prior to PPRV infection (10^4^PFU, *i.p*). **(B)** Body weight was monitored for each group and is shown in line graph. **(C)** Bar diagrams represent the virus titers that was measured from the brain tissues at the termination of the experiment. **(D**, **E)** Representative FACS plots for CD8^+^CD44^+^ T cells in the spleens of IFNR KO mice from different groups and the frequencies of these cells are shown by bar diagrams. **(F–H)** CFSE labeled transferred cells were detected in the spleens of infected recipient animals. Representative histogram plots show the extent of CFSE dilution in total CD8^+^ cells in the spleens of recipient animals **(F)**. Bar diagrams represent the frequencies **(G)** and counts **(H)** of CFSE diluted cells. Bar diagrams are represented as values ± SEM and the level of statistical significance is determined by either one–way ANOVA or unpaired student t–test and the p–values are defined as p < 0.05 *, p < 0.01 **, p < 0.0001 **** and ns for not significant.

We then tested whether or not the expanded cells in peptide immunized mice had anti–PPRV activity. The LN cells isolated from peptide immunized animals were expanded *ex vivo* for three days by stimulating with the peptides. Thereafter, the cells were labeled with CFSE and transferred into IFNR KO mice that were then challenged *i.p* with PPRV (**Figure 8A**). IFNR KO mice receiving peptide–pulsed cells reduced their body weights to a lesser extent as compared to those not receiving such cells (**Figure 8B**). The animals were sacrificed at 4 dpi to analyze cellular response and PPRV loads in brain tissues. Higher frequencies of CD8^+^ T cells expressed CD44 in the peptide pulsed recipients as compared to the other groups that suggested for their activation (**Figures 8D, E**). The frequencies and the numbers of cells that diluted CFSE were two–fold higher in the animals receiving the peptide–pulsed cells as compared to those receiving un–pulsed cells (**Figures 8F–H**). A slight but significant reduction in the viral titers of animals receiving unpulsed cells was observed but animals receiving PPRV–peptide pulsed cells showed up to a 100–fold reduction in the replicating PPRV titers (**Figure 8C**). These results clearly showed the functionality of PPRV–specific CD8^+^ T cells expanded by peptide immunization.

We identified class I MHC restricted peptides of PPRV. The peptide– immunized animals activated and expanded PPRV–specific CD8^+^ T cells and such cells helped control PPRV infection as shown by an adoptive transfer approach.

## Discussion

With the ramped–up efforts to eradicate PPRV by intensive vaccination programs in many countries, it has become imperative to develop an accessible laboratory animal model to better understand the viral pathogenesis and more importantly discovering the immune correlates of protection against the virus. Such analysis could enhance the prospects of devising alternative vaccine strategies as morbilliviruses are known to cause immunosuppression. We, therefore, undertook this study to develop a laboratory mouse model not only to elucidate events in PPRV pathogenesis but also to decipher the role of innate immune cells as well as the virus–specific cytotoxic T cells during the viral infection. IFNR KO mice exhibited susceptibility to PPRV infection and permitted the virus replication in most of the organs. Neutrophils and macrophages likely served as the Trojan horse and transported the virus to CNS leading to the virus induced encephalitic lesions. The expanded CD8^+^ T cells and NK cells correlated with the protection of PPRV infected WT mice. In order to analyze PPRV–specific CD8^+^ T cell response, we discovered H–2K^b^ restricted immunogenic epitopes of PPRV and enumerated the virus–specific CD8^+^ T cells in infected as well as immunized WT C57BL/6 mice using an array of class I MHC tetramers generated by a throughput tetramer technology. We consider that the study could pave the way for investigating PPRV pathogenesis in a more accessible laboratory model to further elucidate protective role of anti–viral CD8^+^ T cells.

PPRV, a member of morbillivirus genus in paramyxoviridae family, incurs significant losses to animal husbandry sector in many countries due to its endemicity (30). Therefore, intensive vaccination programs are being adopted in many countries to eradicate the virus. A laboratory mouse model would be useful not only to better understand the contribution of cellular and molecular mediators in the viral pathogenesis but also to test the efficacy of anti–virals. Many members of genus morbillivirus are neurovirulent and PPRV was shown to induce neurovirulence in naturally infected goat neonates (27, 28). Therefore, the infectivity of IFNR KO mice by PPRV, the observed neurovirulence as well as infiltration of immune cells in infected tissues could suggest that this model could represent a better accessible model for deciphering molecular and cellular mechanisms during the virus pathogenesis. We observed that PPRV pulsed innate immune cells facilitated the transport of PPRV to CNS and donor neutrophils and macrophages were abundantly recovered from brain tissues of recipient mice. Although we did not recover significantly higher frequencies of donor DCs (CD45.1^+^CD11b^+^CD11c^+^) in the brain tissues of recipient mice but the disease severity was comparable in all the recipients (**Figures 3B–D**). Several factors could explain these results such as the inability of transferred DCs to directly home to brain tissues or their inefficient proliferation. Nonetheless, such cells might have transferred the virus to other inflammatory cells, which in turn could have transported it to brain tissues and elsewhere. The observed increased leukocytic infiltration in the brain tissues supported this notion (**Figure 3C** and data not shown).

A class of host–derived molecules with potent anti–viral effects are type I IFNs that acts both in autocrine and more efficiently in a paracrine manner to induce in the responding cells an anti–viral state (31, 32). We observed distinct expression patterns of type I IFNs in PPRV stimulated innate immune cells of murine origin and such responses could be attributed to the dose of the virus or the intrinsic properties of such cells (RAW macrophages versus bone marrow derived primary macrophages) (**Figure S1**). However, the results could suggest for diversified functions associated with IFNα and IFNβ. Such a phenomenon is well documented for the activity of type I and type III IFNs and was largely attributed to the expression pattern of their receptor subunits (33). A dichotomy is also known for the activity of type I IFNs (IFNα and IFNβ) (34). Furthermore, a specific inhibition of IFNβ signaling by antibodies changed the course of a persistent viral infection (LCMV clone 13) into an infection that was efficiently controlled in the acute phase (35). Therefore, further analysis to decipher the relative roles of different species of type I IFNs in the protection against PPRV could be worth investigating.

In WT animals the activity of NK cells might have controlled PPRV infection and T cells could have played a subsidiary antiviral role (**Figure 4**). What properties of NK cells are critical? While this is a pertinent question but was not investigated further in the current communication. Their efficient infiltration both in BAL and lung tissues of IFNR KO mice could nonetheless suggest their anti–PPRV activity. However, the recruited cells failed to protect IFNR KO mice against PPRV suggesting a crucial role of IFN signaling in NK cells for an efficient anti–viral activity and that their killing activity alone might not sufficiently help achieve the viral control (**Figures 4F, G**). Furthermore, the antigen presenting cells stimulated both CD4^+^ and CD8^+^ T cells but the function of such cells could have been compromised due to their deficiency of type I IFN response. The expansion and migration of T cells in PPRV infected WT mice compared to those from IFNR KO mice could also indicate the critical role of IFN signaling in activation or migration. CD4^+^ T cell responses precede CD8^+^ T cells was shown earlier (30). It was also suggested that an inefficient JAK/STAT signaling following type I IFN ligation enhances the propensity of CD8^+^ T cells to undergo apoptosis by host factors such as the glucocorticoids. Multiple studies have shown that microbial infections can actively engage hypothalamic pituitary adrenal (HPA) axis to induce glucocorticoids that can induce apoptosis of CD8^+^ T cells (22). Whether or not PPRV infection predominantly activate HPA axis is not known. That the CD8^+^ T cells could be involved in anti–PPRV immunity was shown by adoptive transfer of WT CD8^+^ T cells, or those isolated from previously infected as well as from PPRV peptides immunized mice (**Figures 5**, **8**). Therefore, vaccine induced enhancement of PPRV specific CD8^+^ T cell responses could be perceived as an efficient strategy to control PPRV. We observed an expansion of a population of CD8^+^ T cells that expressed both CXCR3 and high levels of CD62L in WT animals only and such a phenotype was conspicuously absent in IFNR KO mice despite their having abundant PPRV antigens (**Figure S4**). Such a phenotype was shown to have potent effector functions (25, 26). These observations could also suggest for the induction of distinct pathways in differentiating CD8^+^ T cells are crucially dictated by IFN signaling.

With limited data available for PPRV cell and tissue tropism, the virus replication was demonstrated in the lymphoid organs (6). The known receptor for PPRV are SLAM family proteins (36). Our results demonstrated that the cells of innate immune origin expanded in PPRV infected animals were susceptible to the viral infection. Surprisingly innate immune cells such as neutrophils and macrophages were potentially able to transfer virus to distal locations (**Figures 2**, **3**). The innate immune cells express SLAM receptors abundantly and therefore their susceptibility to PPRV infection could involve these receptors (36). A particular receptor to enhance the susceptibility of mice was not been identified in this communication and constitutes part of our ongoing investigations. It would be interesting to explore whether PPRV replicates in the innate immune cells of sheep and goats as well. If indeed true, it might necessitate revisiting vaccination strategies to help achieve a complete viral eradication. Neurovirulence and neuropathology induced by PPRV are recently reported in goat kids and newly born BALB/c mice as well as CD1d knockout mice, the latter induce inefficient NK cells responses (37, 38). However, the susceptibility of IFNR KO mice to PPRV was not shown earlier. Our observations could be more relevant as within some herds of goats, mutations in one or more components of signaling pathways involving type I IFNs could contribute to the unabated spread of PPRV. Animals and humans with signaling defects in pathways leading to type I IFN production are exceedingly susceptible to viral infections (31, 39). We used a vaccine strain of PPRV that induced a hundred percent mortality in IFNR KO mice and no apparent disease in WT C57BL/6 mice. Whether or not the virulent strain of PPRV can induce the disease and trigger potent CTL response in WT mice is currently being investigated in our laboratory.

An initial encounter of host with viruses elicit type I IFN response, but for a long term protection the optimal activity of CD8^+^ T cells is crucial in virus clearance (22, 40). This necessitates identifying class I epitopes that can be used to quantify and assess functionality of such cells (41),. Although, some studies have suggested for a limited role of CD8^+^ T cells in vaccinated animals in providing protection against PPRV infection, peptides that could efficient expand antigen–specific CD8^+^ T cells response are yet to be discovered (5, 42–44). Designing of a vaccine against an intracellular pathogen requires identifying immunogenic epitopes that can also serve as subunit vaccine candidates. Screening of peptides of an antigen is best done by *in silico* analysis as invariably a large number of linear amino acid sequences need to be probed. The utility of class I MHC tetramers in staining antigen–specific CD8^+^ T cells and discovering epitopes in a throughput manner hold unmatched potential but has not been adequately put to use particularly for animal pathogens (23, 45). The prediction of peptide is necessary for diagnosis, formulating vaccines as well as for analyzing the functionality of cells (23, 41, 46, 47). We discovered at least four immunogenic epitopes from structural proteins of PPRV using *in silico, ex vivo* and *in vivo* approaches. That the immunization of mice with a cocktail of peptides induced PPRV specific CD8^+^ T cells constitute first such example for PPRV. Furthermore, such an approach provides impetus to subunit vaccine development. Our ongoing investigations are aimed at deciphering pathways in differenting PPRV–specific CD8^+^ T cells.

## Data Availability Statement

The datasets presented in this study can be found in online repositories. The names of the repository/repositories and accession number(s) can be found in the article/**Supplementary Material**.

## Ethics Statement

The animal study was reviewed and approved by Institutional Animal Ethics Committee, IISER Mohali, constituted under the aegis of committee for the purpose of control and supervision of experiments on animals (CPCSEA).

## Author Contributions

YS and RS contributed equally to the work. All authors contributed to the article and approved the submitted version.

## Funding 

The study was supported by extramural grant from National Agriculture Science Fund (NASF/ABA–6021/2017–18).

## Conflict of Interest

The authors declare that the research was conducted in the absence of any commercial or financial relationships that could be construed as a potential conflict of interest.
