# Supplementary material for: A Mouse Model of PPRV Infection for Elucidating Protective and Pathological Roles of Immune Cells
Source: Front Immunol. 2021 Apr 12;12:630307. doi: 10.3389/fimmu.2021.630307 (PMC8072281; doi:10.3389/fimmu.2021.630307)
Supplement: Supplementary file 1 [file DataSheet_1.pdf]

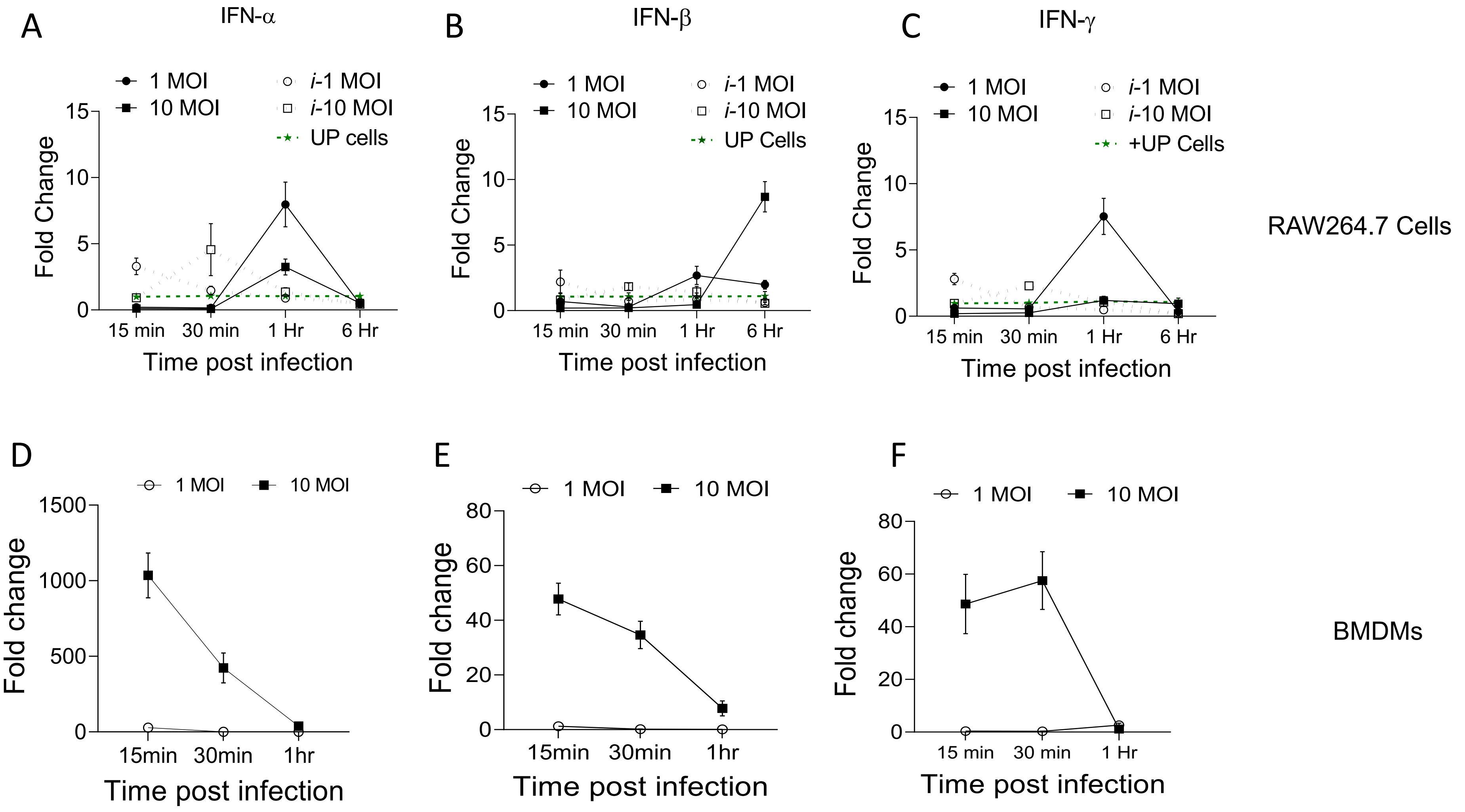

Figure S1. Murine macrophages (RAW macrophages and primary bone marrow derived macrophages, BMDMs) were pulsed with live PPRV or heat inactivated PPRV (*i*-PPRV) at low (1) and high (10) multiplicity of infections (MOI) to measure IFNα, IFNβ and IFNγ response. The PPRV exposed cells were collected at different times to measure the expression of different IFNs by qRT-PCR. A-C. Fold change in interferon expression in RAW264.7 cells upon infection with live PPRV (continuous line plot), heat inactivated PPRV (dotted line plot) and for unpulsed cells (UP) green dashed line plot. D-F. Line plots show fold change expression of BMDMs upon live PPRV infection. *(i-* heat inactivated PPRV; UP, unpulsed cells).

Gating controls for different populations

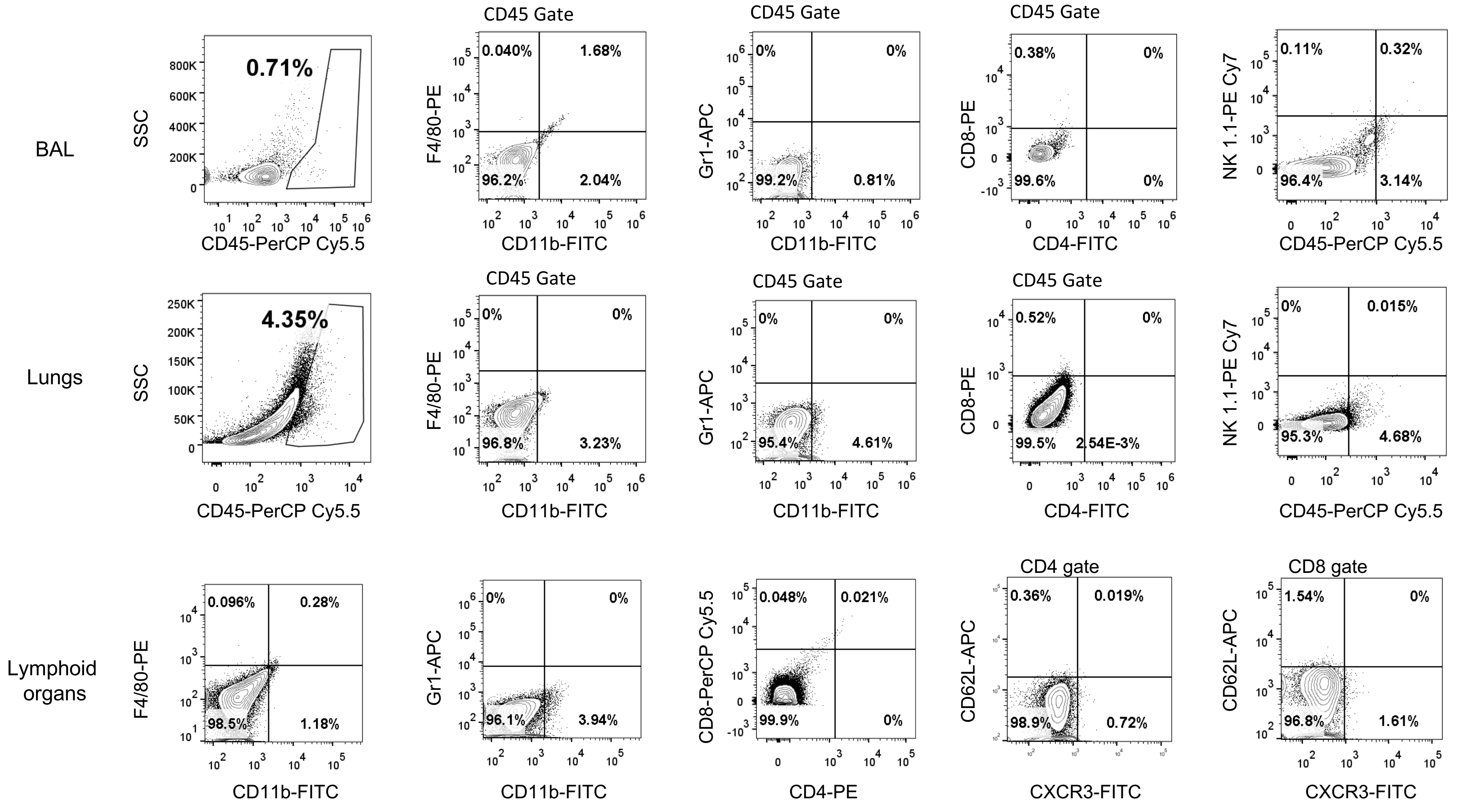

Figure S2. Gating strategy for analyzing different immune cells in lymphoid and non-lymphoid organs. Different antibody combinations were used for measuring the distribution of innate immune cells and T cells. To measure cells in non-lymphoid organs such as BAL and lungs, $CD45^+$ cells were first gated and then opened for the indicated cell surface markers. For NK cells, live cells were gated and opened for CD45 on one axis (x) and NK1.1 on the other axis (y). While measuring immune cells in lymphoid organs, live cells gate were opened for different cell surface markers as indicated in FACS plots.

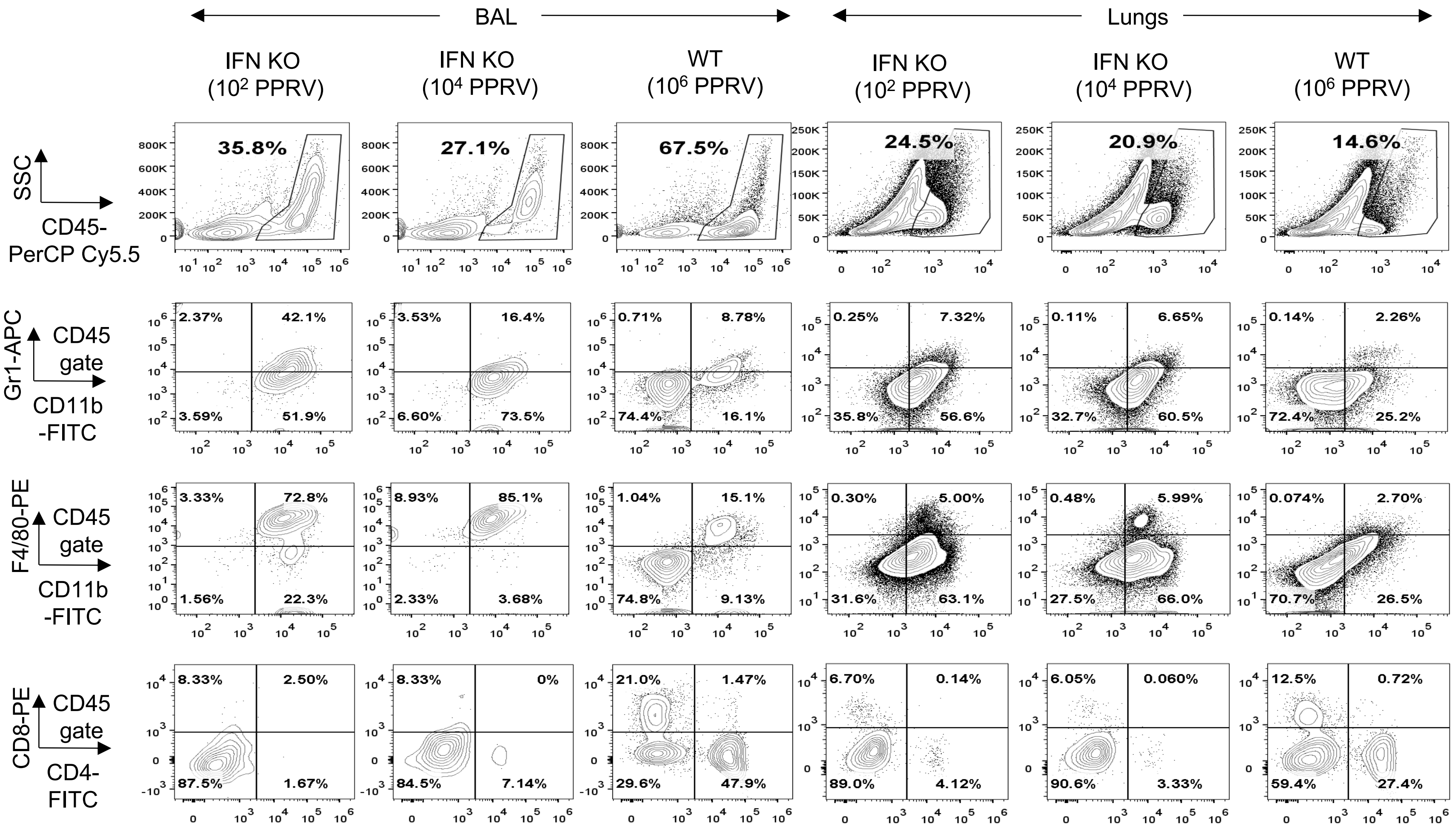

Figure S3. Representative FACS plots show the cellular distribution in bronchoalveolar lavage (BAL) and lung tissues of PPRV infected IFNR KO mice. The animals were infected with the indicated doses of PPRV via intranasal route and scarified on 6dpi and the cellular analyses were performed in BAL and lung tissues. Indicated markers are shown in the respective FACS plots.

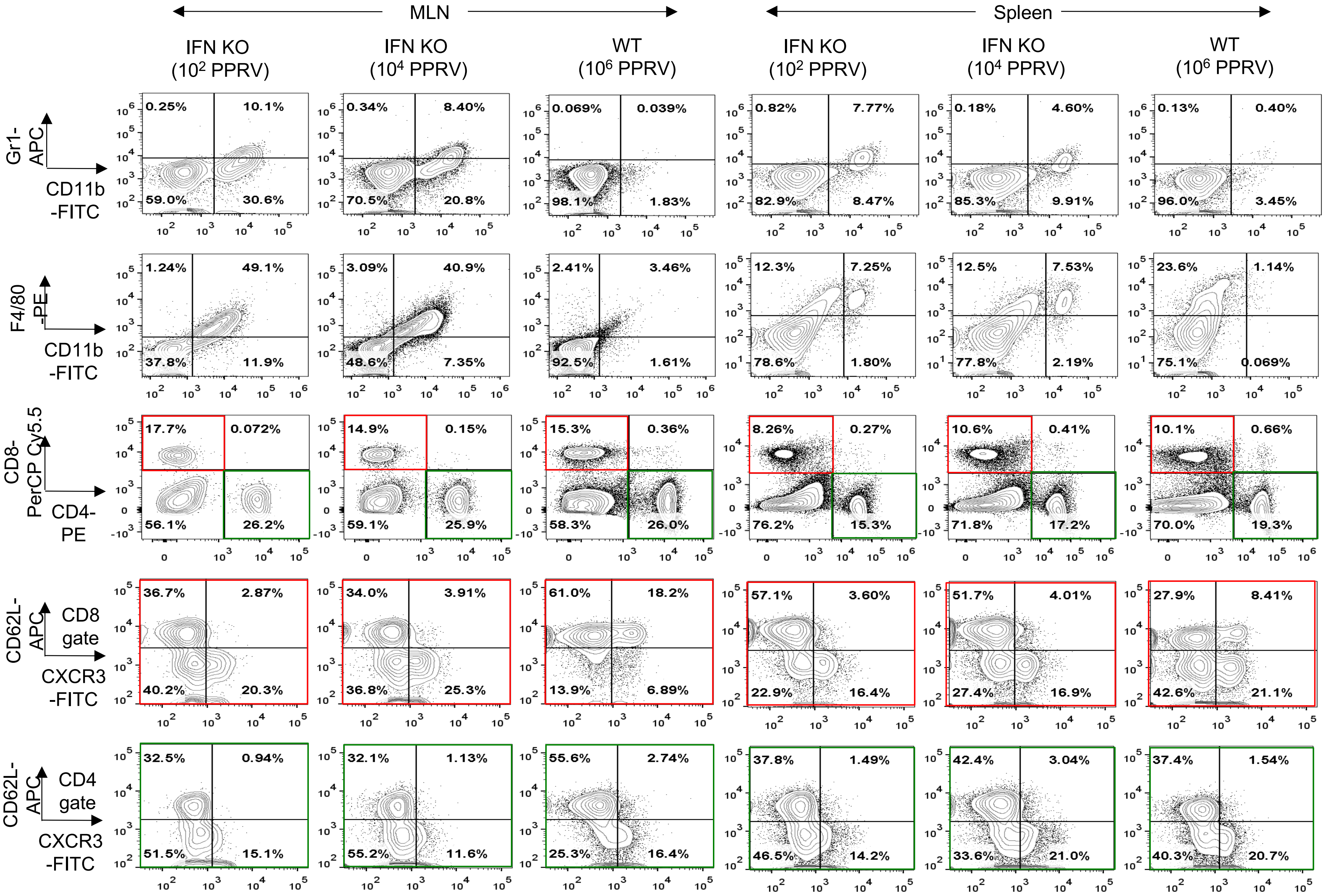

Figure S4. Representative FACS plots show the cellular distribution in mediastinal lymph nodes (MLN) and spleen of PPRV infected IFNR KO mice. The animals were infected with the indicated doses of PPRV via intranasal route and scarified on 6dpi and the cellular analyses were performed in single cell suspension of MLN and spleen. Indicated markers are shown in the respective FACS plots.

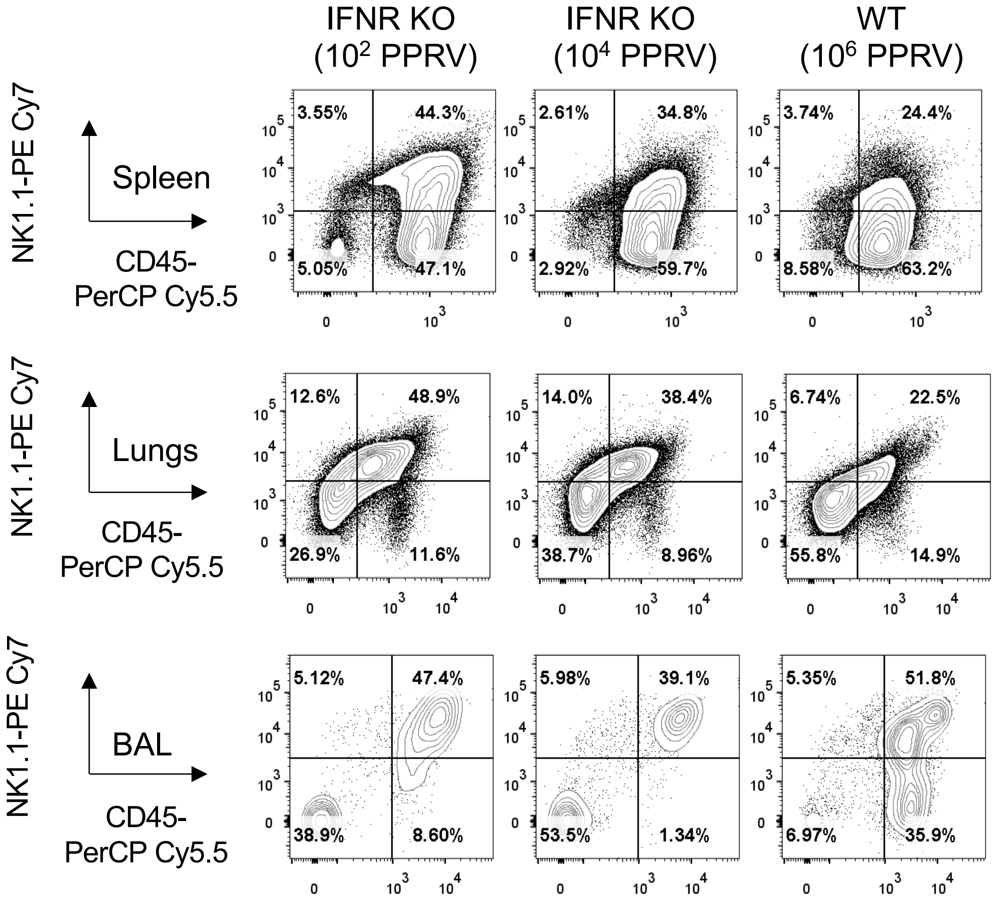

Figure S5. Representative FACS plots show the distribution of NK cells in indicated organs of PPRV infected IFNR KO mice. The animals were infected with the indicated doses of PPRV via intranasal route and scarified on 6dpi. The cellular analyses were performed in single cell suspension of spleen, lungs and BAL. Indicated markers are shown in the respective FACS plots.

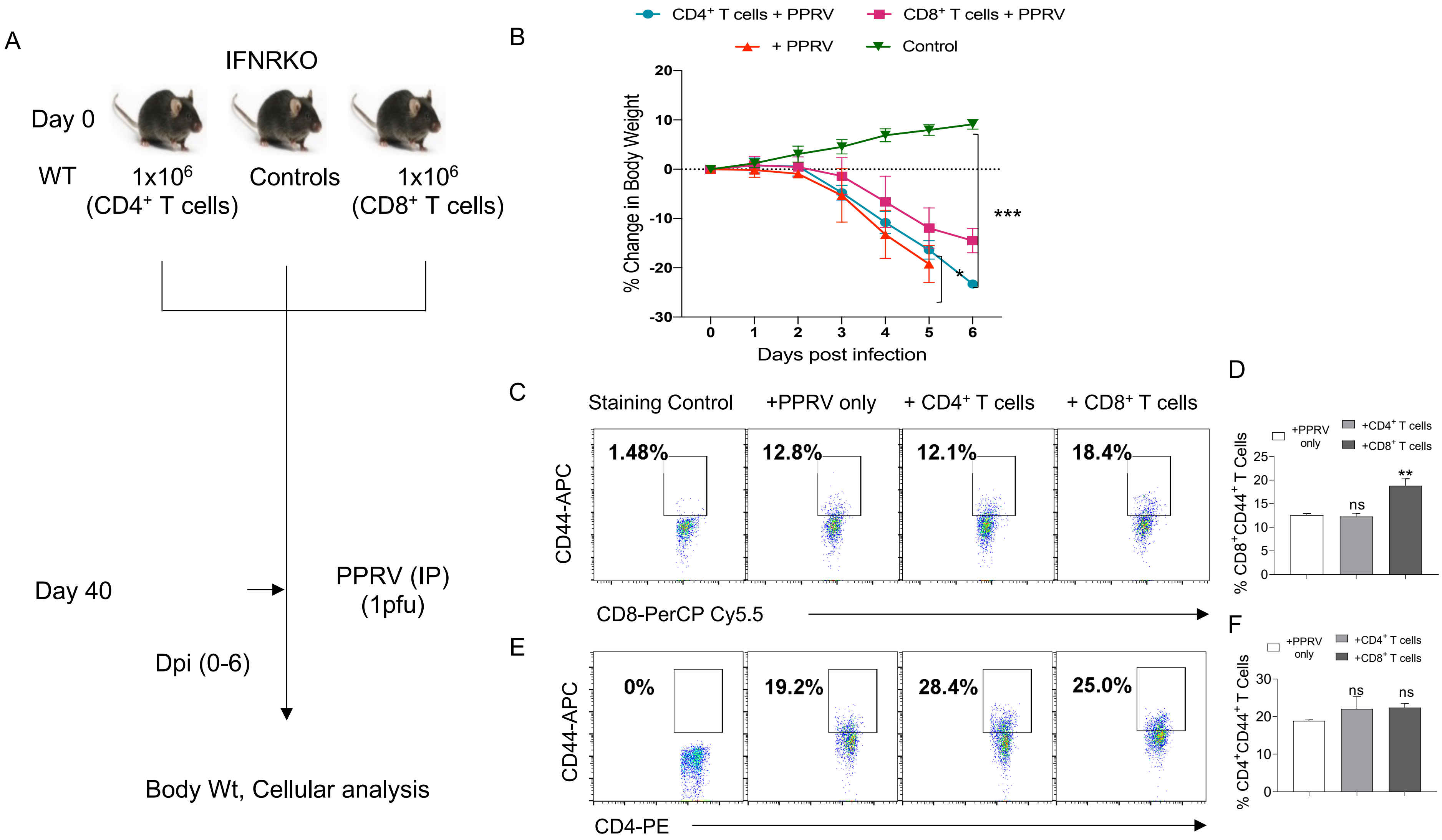

Figure S6. WT CD8⁺ T cells delay morbidity in PPRV infected IFNR KO mice. A. A schematic of the experiments is shown. 1x10⁶ CD4⁺ or CD8⁺ T cells from WT mice (CD45.1⁺) into IFNR KO mice (CD45.2⁺). After 40 days, animals were *i.p* infected with PPRV (n=4/group). The disease progression and the activation profile of CD4⁺ and CD8⁺ T cells were measured. B. Percent change in body weight of mice from each group is shown. The level of statistical significance was determined by one-way ANOVA test. C. Representative FACS plots show the activation of CD8⁺ (C) and CD4⁺ T cells (E) recovered from the spleen samples in different groups. D and F. In each group four animals were used. Cumulative data on the activation profile of CD8⁺ and CD4⁺ T cells respectively is shown by bar diagrams. Student t test was used for analysis. The experiments were repeated two more times with essentially similar results. $p < 0.05$ *, $p < 0.01$ **, $p < 0.001$*** and ns for not significant.

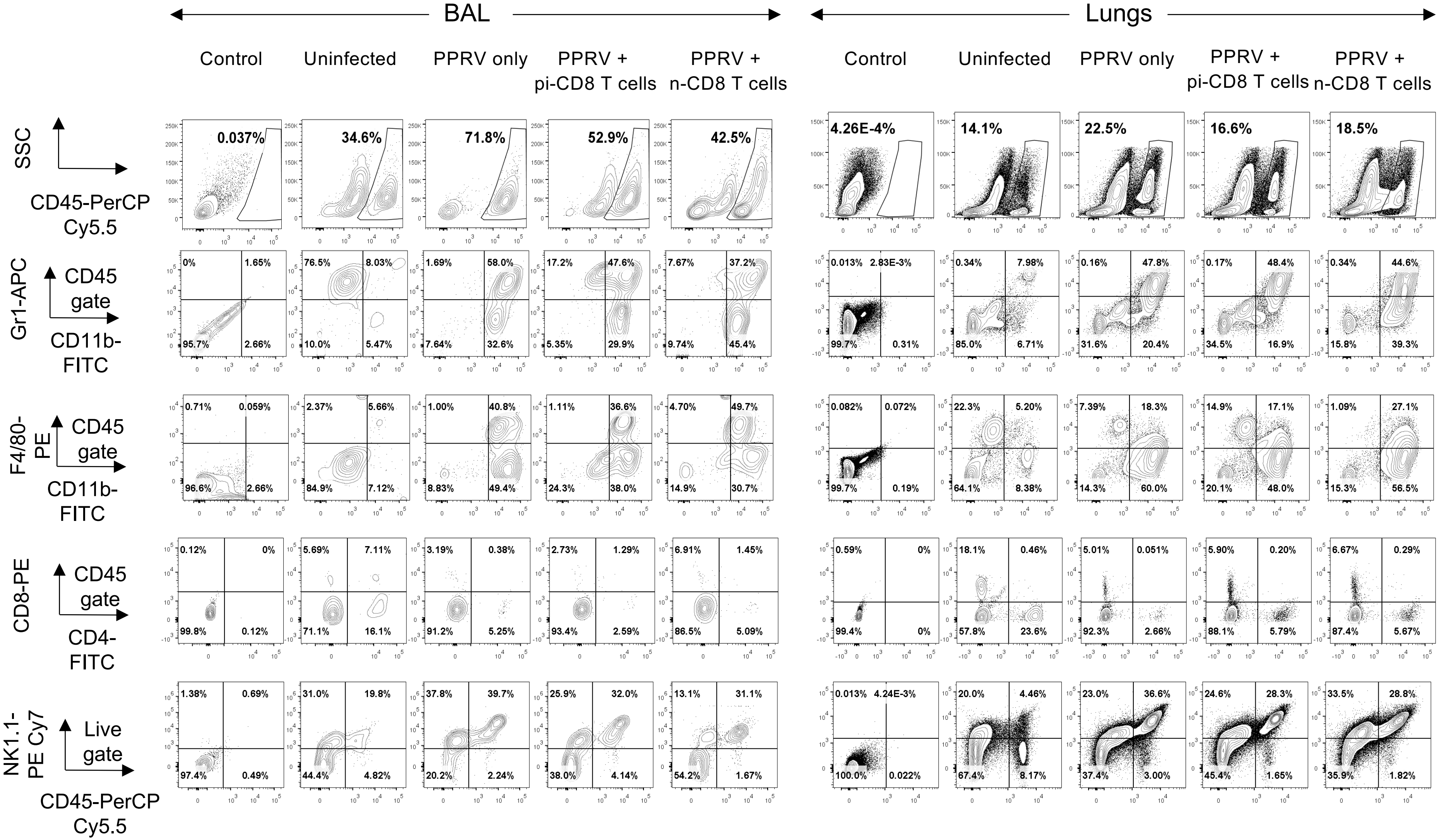

Figure S7. Representative FACS plots show the distribution of immune cells in single cell suspensions prepared from BAL and lungs of of PPRV infected IFNR KO mice transferred with WT CD8⁺ T cells collected from naïve or the previously PPRV infected mice. The control and recipients were infected with the 10^4 of PPRV via intranasal route and scarified on 6dpi. A. The cellular analyses were performed in single cell suspension of BAL and lungs. Indicated markers are shown in the respective FACS plots

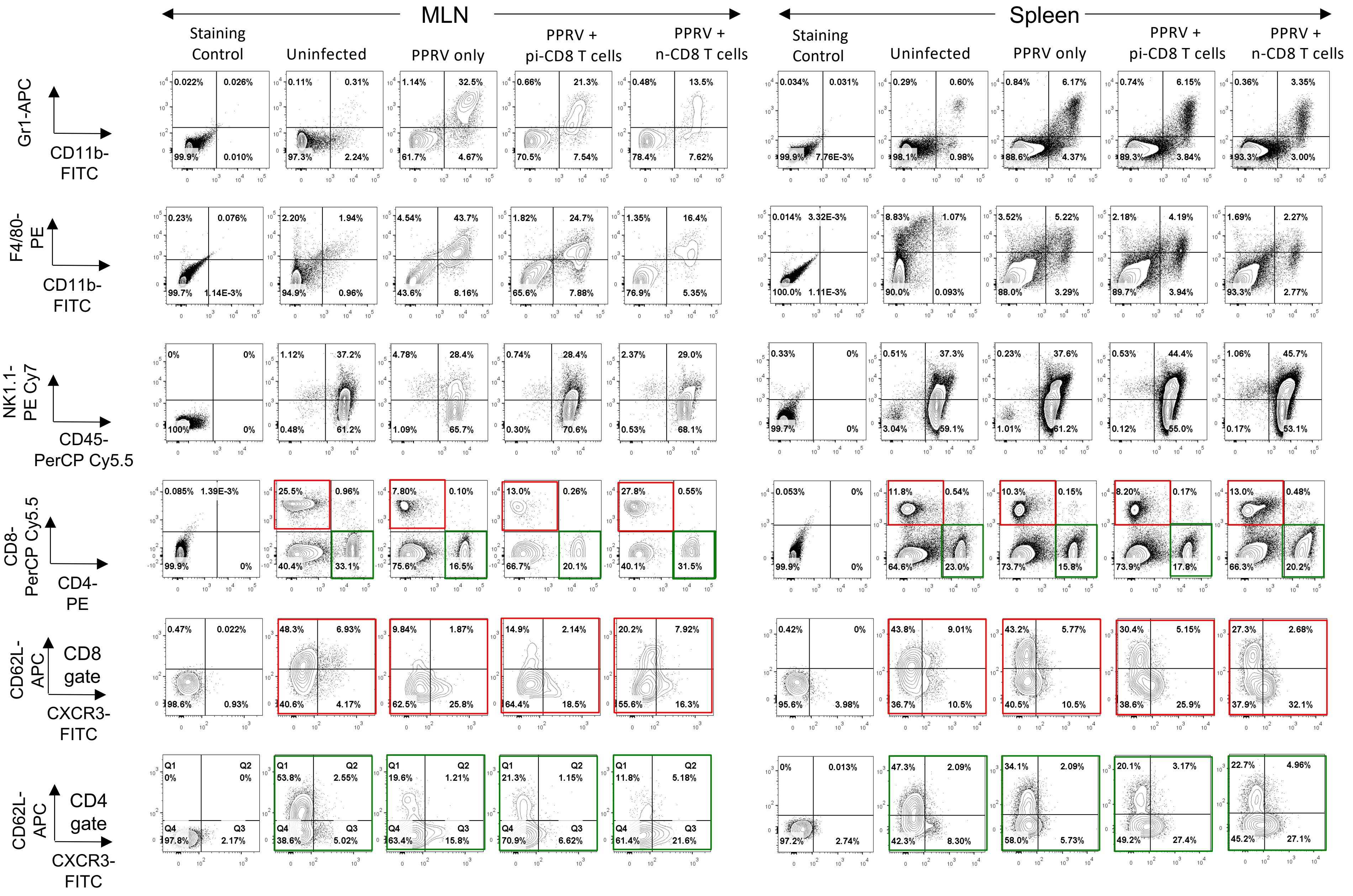

Figure S8. Representative FACS plots show the distribution of immune cells in single cell suspensions prepared from MLN and spleen of of PPRV infected IFNR KO mice transferred with WT CD8^{+} T cells that were collected from naïve or the previously PPRV infected mice. The control and recipients were infected with the 10^4 of PPRV via intranasal route and scarified on 6dpi.

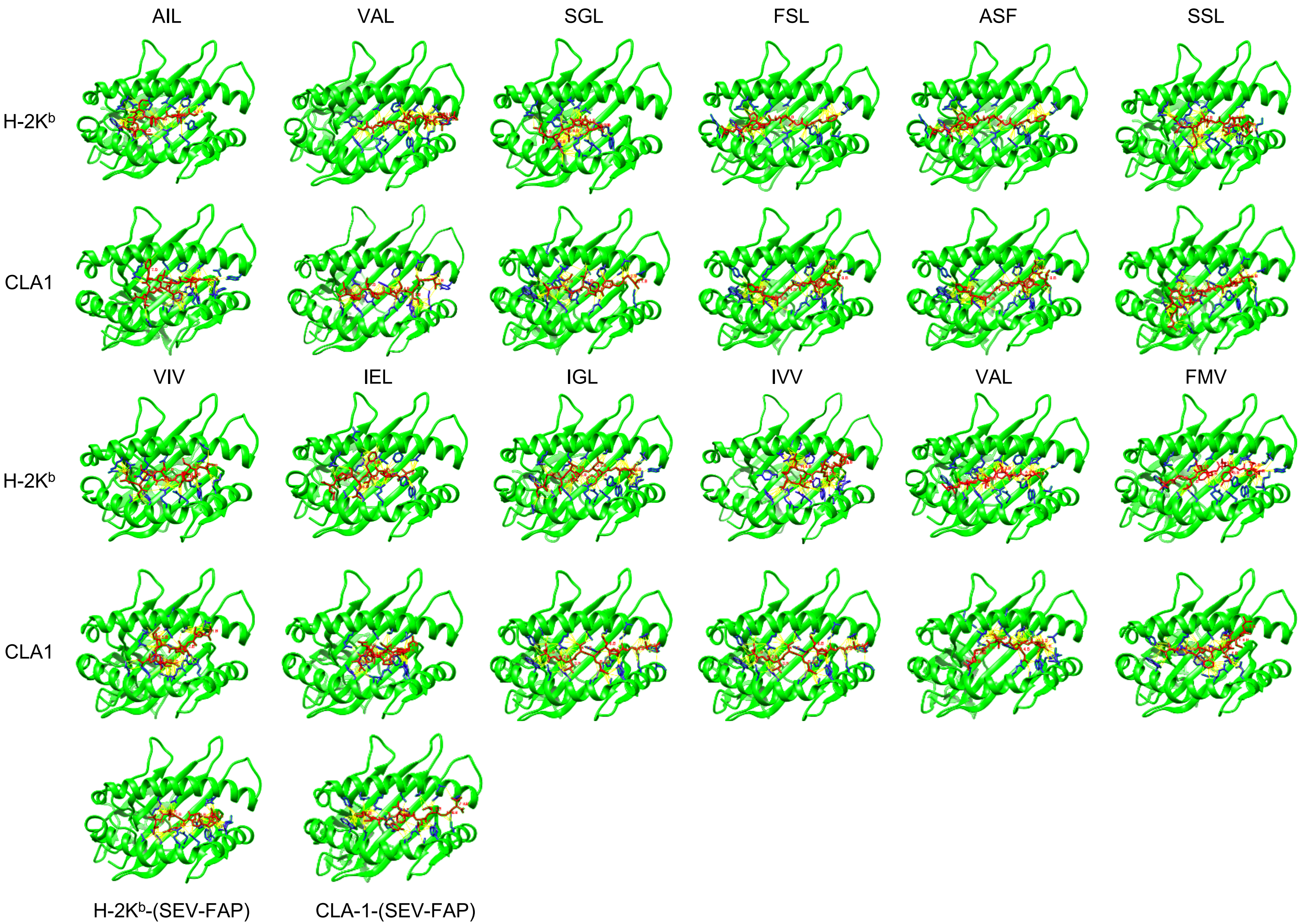

Figure S9. Molecular docking of predicted PPRV peptides for class I MHC molecule of mouse (H-2K^{b}) and goat (CLA-1). Top view of MHC-I (alpha chain) is shown green, PPRV peptide in red and interacting residues of MHC-I are shown in blue, while interactions are shown in yellow.

Table S1. RT-qPCR primers used for measuring type I IFN response

Gene name	Forward primer	Reverse primer	Product Size
mIFN-α	5'-GGACTTTGGATTCCCGCAGGA-3'	5'- GTGGAGGTCATTGCAGAATGAG-3'	169
mIFN-β	5'-CCAGCTCCAAGAAAGGACGA-3'	5'-CGCCCTGTAGGTGAGGTTGAT-3'	105
mIFN-γ	5'-TTCTTCAGCAACAGCAAGGC-3'	5'-TCAGCAGCGACTCCTTTTCC-3'	165
HPRT	5'-CTGGAGCAAGTCTTACAGTCC-3'	5'-GCGTCGTGATTAGCCATGATG-3'	135

Table S2. A list of top scoring H-2K^b restricted nonameric peptides

Nucleocapsid

Peptide	Length	Score
ASFILTIKF	9	0.18398
SSIITRSRL	9	0.15426
FSAGAYPLL	9	0.06558
SIITRSRLL	9	0.01413
SLRRFMVSL	9	-0.0442
STIESLMNL	9	-0.1796
SLMNLYQQL	9	-0.2432
INGSKLTGV	9	-0.3234
ATLLKSLAL	9	-0.367
VMISMLSLF	9	-0.4508

Matrix

Peptide	Length	Score
ANAVAFNFL	9	0.26548
IVVRRTAGL	9	0.20524
VAFNFLVTL	9	0.19257
FMYLFLLGV	9	0.10164
TFMVHVGNF	9	0.07972
CNAVNLVPL	9	0.06586
LVFYNNTPL	9	0.04817
VFYNNTPLS	9	0.00669
ECFMYLFLL	9	-0.0604
VVYMSITRL	9	-0.1507

Fusion

Peptide	Length	Score
SGGDFLAIL	9	0.24766
VAILTFLYL	9	0.16852
AILTFLYLF	9	0.12942
FLYLFPNAV	9	0.1086
VSLGLVTLI	9	0.08484
FGGNMYIAL	9	-0.0348
SVYLHKIDL	9	-0.0586
IAYPTLSEI	9	-0.0661
SVHRMSCEL	9	-0.2512
ALYPMSPLL	9	-0.3549

Heamagglutinin

Peptide	Length	Score
IEHIFESPL	9	0.20657
VYYIYDTGL	9	0.20256
VILDRERLV	9	0.20057
VIERPYILL	9	0.17614
IGLVRDLGL	9	0.11966
VTRAHFSEL	9	0.11675
SSYYYPVRL	9	0.04612
VITSVFGPL	9	0.01865
VMFLSLIGL	9	-0.0126
VLLVMFLSL	9	-0.1289

Phosphoprotein

Peptide	Length	Score
SIMIAIPGF	9	0.25074
SEYEYEDDL	9	0.22188
QVQRYYVYS	9	0.04008
ISGATQAVL	9	0.03627
VYLSPEDNL	9	-0.0717
LNIDHKDYL	9	-0.0894
QAYHVNKGL	9	-0.0967
SILLLKGEV	9	-0.1415
INQSCSPAI	9	-0.3988
RSIIKSSKL	9	-0.4542

Large polymerase

Peptide	Length	Score
VFYLTFELV	9	0.21366
TQYVFYLTF	9	0.16418
IQYFRESLL	9	0.11515
VAILEYSGI	9	0.00624
TNFIYQQGM	9	-0.0187
SSFDPYNMI	9	-0.062
LAYPRYSNF	9	-0.1088
YNYLRCQPI	9	-0.117
VIDQRYSEL	9	-0.1436
LNYLNMSRL	9	-0.2933

Table S3: Characteristics of PPRV peptides binding with Class I MHC of mice (H-2K^b)

S.No.	Peptide	Derived from	Sequence	Docking Parameter's					
				I	II	III	IV	V	VI
**1**	**AIL**	**Fusion Protein**	**AILTFLFLL**	**-191.11**	**Yes**	**Yes**	**No**	**Yes**	**Yes**
2	VAL	Fusion Protein	VAILTFLFL	-209.927	Yes	No	No	No	Yes
3	SGL	Fusion Protein	SGGDFLAIL	-179.766	No	No	Yes	No	No
**4**	**FSL**	**Nucleocapsid Protein**	**FSAGAYPLL**	**-220.2**	**Yes**	**Yes**	**No**	**No**	**Yes**
5	ASF	Nucleocapsid Protein	ASFILTIKF	-194.624	Yes	No	Yes	Yes	Yes
6	SSL	Nucleocapsid Protein	SSITTRSRL	-187.869	No	No	Yes	Yes	No
7	VIV	Matrix Protein	VILDRERLV	-160.121	No	No	No	No	No
8	IEL	Hemagglutinin Protein	IEHIFESPL	-173.187	No	No	Yes	No	No
**9**	**IGL**	**Hemagglutinin Protein**	**IGLVRDFGL**	**-176.27**	**Yes**	**Yes**	**Yes**	**Yes**	**Yes**
10	IVV	Matrix Protein	IVVRRTAGV	-194.463	Yes	No	Yes	No	Yes
11	VATL	Matrix Protein	VAFNILVTL	-208.709	Yes	No	Yes	No	No
**12**	**FMV**	**Matrix Protein**	**FMYLFLLGV**	**-243.288**	**Yes**	**No**	**Yes**	**No**	**Yes**
13	SEV	Sendai Virus	FAPGNYPAL	-233.646	Yes	Yes	Yes	Yes	Yes

I. Docking energy scores, II. N-terminus of the docked peptides should be deeply embedded into H2K^b while C- terminal is held by salt-bridge near the surface of H-2K^b. III. Residues 4 or 5 of the docked peptides may bulge out of the groove and do not make Vander Waal's contact with H-2K^b. IV. Residue 5 or 6 are most probable primary anchor residue V. Residues at position 8 or 9 may act as secondary anchors. VI. Polar interactions (H-bonds) at peptide termini with H-2K^b stabilize its docking.

Table S4: Characteristics of PPRV peptides binding with Class I MHC of goat CLA-1

S.No.	Peptide	Derived from	Sequence	Docking Parameter's					
				I	II	III	IV	V	VI
**1**	**AIL**	**Fusion Protein**	**AILTFLFLL**	**-220.883**	**Yes**	**Yes**	**Yes**	**No**	**Yes**
2	VAL	Fusion Protein	VAILTFLFL	-234.772	No	Yes	No	Yes	Yes
3	SGL	Fusion Protein	SGGDFLAIL	-193.167	Yes	No	No	Yes	Yes
**4**	**FSL**	**Nucleocapsid Protein**	**FSAGAYPLL**	**-234.857**	**Yes**	**Yes**	**Yes**	**No**	**Yes**
5	ASF	Nucleocapsid Protein	ASFILTIKF	-237.75	Yes	Yes	No	No	Yes
6	SSL	Nucleocapsid Protein	SSITTRSRL	-206.182	No	No	No	Yes	No
7	VIV	Matrix Protein	VILDRERLV	-175.016	No	No	No	No	No
8	IEL	Hemagglutinin Protein	IEHIFESPL	-211.283	No	Yes	Yes	No	No
**9**	**IGL**	**Hemagglutinin Protein**	**IGLVRDFGL**	**-221.898**	**Yes**	**Yes**	**Yes**	**No**	**No**
10	IVV	Matrix Protein	IVVRRTAGV	-204.106	No	Yes	Yes	Yes	Yes
11	VATL	Matrix Protein	VAFNILVTL	-228.412	No	No	Yes	No	Yes
**12**	**FMV**	**Matrix Protein**	**FMYLFLLGV**	**-262.773**	**Yes**	**Yes**	**Yes**	**No**	**No**
13	SEV	Sendai Virus	FAPGNYPAL	-258.567	Yes	Yes	Yes	Yes	Yes

I. Docking energy score, II. N terminus of the docked peptides should be deeply embedded into CLA-1 while the C- terminus is held by salt-bridge near the surface of CLA-1. III. Residue 4 or 5 of the docked peptides may bulge out of groove and do not make Vander-Waal's contacts with CLA-1. IV. Residue 5 or 6 are the most probable primary anchor residue V. Residues at position 8 or 9 may act as secondary anchors. VI. Polar interactions (H-bonds) at peptide termini with CLA-1 stabilize the docking.
